# Modified forward-backward splitting midpoint method with superposition perturbations for the sum of two kinds of infinite accretive mappings and its applications

**DOI:** 10.1186/s13660-017-1506-9

**Published:** 2017-09-18

**Authors:** Li Wei, Liling Duan, Ravi P Agarwal, Rui Chen, Yaqin Zheng

**Affiliations:** 1 School of Mathematics and Statistics, Hebei University of Economics and Business, 47 Xuefu Road, Shijiazhuang, China; 2 Department of Mathematics, Texas A&M University-Kingsville, Kingsville, TX 78363 USA; 3 College of Science, Agricultural University of Hebei, Baoding, China

**Keywords:** *p*-uniformly smooth Banach space, $\theta_{i}$-inversely strongly accretive mapping, $\gamma_{i}$-strongly accretive mapping, $\mu_{i}$-strictly pseudo-contractive mapping, perturbed operator

## Abstract

In a real uniformly convex and *p*-uniformly smooth Banach space, a modified forward-backward splitting iterative algorithm is presented, where the computational errors and the superposition of perturbed operators are considered. The iterative sequence is proved to be convergent strongly to zero point of the sum of infinite m-accretive mappings and infinite $\theta_{i}$-inversely strongly accretive mappings, which is also the unique solution of one kind variational inequalities. Some new proof techniques can be found, especially, a new inequality is employed compared to some of the recent work. Moreover, the applications of the newly obtained iterative algorithm to integro-differential systems and convex minimization problems are exemplified.

## Introduction and preliminaries

Let *X* be a real Banach space with norm $\Vert \cdot \Vert $ and $X^{*}$ be its dual space. ‘→’ denotes strong convergence and $\langle x,f \rangle$ is the value of $f \in X^{*}$ at $x \in X$.

The function $\rho_{X}: [0,+\infty) \rightarrow [0,+\infty)$ is called the modulus of smoothness of *X* if it is defined as follows:

$$\rho_{X}(t) = \operatorname{sup} \biggl\{ \frac{1}{2} \bigl( \Vert x+y \Vert + \Vert x-y \Vert \bigr)-1: x,y \in X, \Vert x \Vert =1, \Vert y \Vert \leq t \biggr\} . $$

A Banach space *X* is said to be uniformly smooth if $\frac{\rho _{X}(t)}{t} \rightarrow0$, as $t \rightarrow0$. Let $p > 1$ be a real number, a Banach space *X* is said to be *p*-uniformly smooth with constant $K_{p}$ if $K_{p} > 0$ such that $\rho_{X}(t)\leq K_{p}t^{p}$ for $t > 0$. It is well known that every *p*-uniformly smooth Banach space is uniformly smooth. For $p> 1$, the generalized duality mapping $J_{p}: X \rightarrow2^{X^{*}}$ is defined by

$$J_{p}x: = \bigl\{ f \in X^{*}: \langle x, f\rangle= \Vert x \Vert ^{p}, \Vert f \Vert = \Vert x \Vert ^{p-1} \bigr\} , \quad x \in X. $$

In particular, $J: = J_{2}$ is called the normalized duality mapping.

For a mapping $T: D(T) \sqsubseteq X \rightarrow X$, we use $F(T)$ and $N(T)$ to denote its fixed point set and zero point set, respectively; that is, $F(T): = \{x\in D(T): Tx = x\}$ and $N(T) = \{x \in D(T): Tx = 0\}$. The mapping $T: D(T) \sqsubseteq X \rightarrow X$ is said to be

1. non-expansive if
  $$\Vert Tx - Ty \Vert \leq \Vert x-y \Vert \quad \text{for }\forall x,y \in D(T); $$
2. contraction with coefficient $k \in(0,1)$ if
  $$\Vert Tx - Ty \Vert \leq k \Vert x - y \Vert \quad \text{for } \forall x,y \in D(T); $$
3. accretive [1, 2] if for all $x, y \in D(T)$, $\langle Tx - Ty, j(x-y)\rangle\geq0$, where $j(x-y) \in J(x-y)$;
*m*-accretive if *T* is accretive and $R(I+\lambda T) = X$ for $\forall\lambda> 0$;
4. *θ*-inversely strongly accretive [3] if for $\theta> 0$, $\forall x,y \in D(T)$, there exists $j_{p}(x - y) \in J_{p}(x-y)$ such that
  $$\bigl\langle Tx - Ty, j_{p}(x-y) \bigr\rangle \geq\theta \Vert Tx - Ty \Vert ^{p}\quad\text{for }\forall x,y \in X; $$
5. *γ*-strongly accretive [2, 3] if for each $x, y \in D(T)$, there exists $j(x-y) \in J(x-y)$ such that
  $$\bigl\langle Tx - Ty, j(x-y) \bigr\rangle \geq\gamma \Vert x - y \Vert ^{2} $$
  for some $\gamma\in(0,1)$;
6. *μ*-strictly pseudo-contractive [4] if for each $x, y \in X$, there exists $j(x-y) \in J(x-y)$ such that
  $$\bigl\langle Tx - Ty, j(x-y) \bigr\rangle \leq \Vert x - y \Vert ^{2}-\mu \bigl\Vert x - y - (Tx - Ty) \bigr\Vert ^{2} $$
  for some $\mu\in(0,1)$.

If *T* is accretive, then for each $r>0$, the non-expansive single-valued mapping $J_{r}^{T}: R(I+rT)\rightarrow D(T)$ defined by $J_{r}^{T}: = (I+rT)^{-1}$ is called the resolvent of *T* [1]. Moreover, $N(T) = F(J_{r}^{T})$.

Let *D* be a nonempty closed convex subset of *X* and *Q* be a mapping of *X* onto *D*. Then *Q* is said to be sunny [5] if $Q(Q(x)+t(x-Q(x))) = Q(x)$ for all $x \in X$ and $t \geq 0$. A mapping *Q* of *X* into *X* is said to be a retraction [5] if $Q^{2} = Q$. If a mapping *Q* is a retraction, then $Q(z) = z$ for every $z \in R(Q)$, where $R(Q)$ is the range of *Q*. A subset *D* of *X* is said to be a sunny non-expansive retract of *X* [5] if there exists a sunny non-expansive retraction of *X* onto *D* and it is called a non-expansive retract of *X* if there exists a non-expansive retraction of *X* onto *D*.

It is a hot topic in applied mathematics to find zero points of the sum of two accretive mappings, namely, a solution of the following inclusion problem:

1.1 $$ 0 \in(A+B)x. $$

For example, a stationary solution to the initial value problem of the evolution equation

1.2 $$ \frac{\partial u}{\partial t} + (A+B)u, \quad u(0) = u_{0} $$

can be recast as (1.1). A forward-backward splitting iterative method for (1.1) means each iteration involves only *A* as the forward step and *B* as the backward step, not the sum $A+B$. The classical forward-backward splitting algorithm is given in the following way:

1.3 $$ x_{n+1} = (I+r_{n} B)^{-1}(I-r_{n} A)x_{n},\quad n \in N. $$

Some of the related work can be seen in [6–8] and the references therein.

In 2015, Wei et al. [9] extended the related work of (1.1) from a Hilbert space to the real smooth and uniformly convex Banach space and from two accretive mappings to two finite families of accretive mappings:

1.4 $$ \textstyle\begin{cases} x_{0} \in D,\\ y_{n} = Q_{D}[(1-\alpha_{n})(x_{n}+e_{n})],\\ z_{n}= (1- \beta_{n})x_{n} + \beta_{n} [a_{0}y_{n} + \sum_{i = 1}^{N} a_{i} J_{r_{n,i}}^{A_{i}}(y_{n}-r_{n,i}B_{i}y_{n})],\\ x_{n+1}=\gamma_{n} \eta f(x_{n})+(I-\gamma_{n}T)z_{n}, \quad n \in N\cup\{ 0\}, \end{cases} $$

where *D* is a nonempty, closed and convex sunny non-expansive retract of *X*, $Q_{D}$ is the sunny non-expansive retraction of *E* onto *D*, $\{e_{n}\}$ is the error, $A_{i}$ and $B_{i}$ are m-accretive mappings and *θ*-inversely strongly accretive mappings, respectively, where $i = 1,2,\ldots, N$. $T: X \rightarrow X$ is a strongly positive linear bounded operator with coefficient *γ̅* and $f: X \rightarrow X$ is a contraction. $\sum_{m = 0}^{N}a_{m} = 1$, $0 < a_{m} < 1$. The iterative sequence $\{x_{n}\}$ is proved to converge strongly to $p_{0} \in\bigcap_{i = 1}^{N} N(A_{i}+B_{i})$, which solves the variational inequality

1.5 $$ \bigl\langle (T-\eta f)p_{0}, J(p_{0}-z) \bigr\rangle \leq0 $$

for $\forall z\in\bigcap_{i = 1}^{N} N(A_{i}+B_{i})$ under some conditions.

The implicit midpoint rule is one of the powerful numerical methods for solving ordinary differential equations, and it has been extensively studied by Alghamdi et al. They presented the following implicit midpoint rule for approximating the fixed point of a non-expansive mapping in a Hilbert space *H* in [10]:

1.6 $$ x_{1} \in H,\quad x_{n+1}= (1-\alpha_{n})x_{n} + \alpha_{n} T \biggl(\frac {x_{n}+x_{n+1}}{2} \biggr), \quad n \in N, $$

where *T* is non-expansive from *H* to *H*. If $F(T) \neq \emptyset$, then they proved that $\{x_{n}\}$ converges weakly to $p_{0} \in F(T)$ under some conditions.

Combining the ideas of forward-backward method and midpoint method, Wei et al. extended the study of two finite families of accretive mappings to two infinite families of accretive mappings [3] in a real *q*-uniformly smooth and uniformly convex Banach space:

1.7 $$ \textstyle\begin{cases} x_{0}\in D,\\ y_{n} = Q_{D}[(1-\alpha_{n})(x_{n}+e'_{n})],\\ z_{n}= \delta_{n}y_{n} + \beta_{n}\sum_{i = 1}^{\infty}a_{i} J_{r_{n,i}}^{A_{i}}[\frac{y_{n}+z_{n}}{2}-r_{n,i}B_{i}(\frac{y_{n}+z_{n}}{2})] +\zeta_{n} e''_{n},\\ x_{n+1}=\gamma_{n} \eta f(x_{n})+(I-\gamma_{n}T)z_{n}+e'''_{n}, \quad n \in N\cup\{0\}, \end{cases} $$

where $\{e'_{n}\}$, $\{e''_{n}\}$ and $\{e'''_{n}\}$ are three error sequences, $A_{i}: D \rightarrow X$ and $B_{i}: D \rightarrow X$ are m-accretive mappings and $\theta_{i}$-inversely strongly accretive mappings, respectively, where $i \in N$. $T: X \rightarrow X$ is a strongly positive linear bounded operator with coefficient *γ̅*, $f: X \rightarrow X $ is a contraction, $\sum_{n = 1}^{\infty}a_{n} = 1$, $0 < a_{n} < 1$, $\delta_{n} + \beta_{n} +\zeta_{n} \equiv1$ for $n \in N\cup\{ 0\}$. The iterative sequence $\{x_{n}\}$ is proved to converge strongly to $p_{0} \in\bigcap_{i = 1}^{\infty} N(A_{i}+B_{i})$, which solves the following variational inequality:

1.8 $$ \bigl\langle (T - \eta f)p_{0}, J(p_{0} - z) \bigr\rangle \leq0, \quad z \in\bigcap_{i = 1}^{\infty}N(A_{i} + B_{i}). $$

In 2012, Ceng et al. [11] presented the following iterative algorithm to approximate zero point of an m-accretive mapping:

1.9 $$ \textstyle\begin{cases} x_{0} \in X,\\ y_{n} = \alpha_{n}x_{n}+(1-\alpha_{n})J^{A}_{r_{n}}x_{n},\\ x_{n+1}= \beta_{n} f(x_{n})+(1-\beta_{n})[J^{A}_{r_{n}}y_{n} - \lambda_{n}\mu _{n}F(J^{A}_{r_{n}}y_{n})], \quad n \in N\cup\{0\}, \end{cases} $$

where $T: X \rightarrow X$ is a *γ*-strongly accretive and *μ*-strictly pseudo-contractive mapping, with $\gamma+ \mu> 1$, $f: E \rightarrow E $ is a contraction and $A: X \rightarrow X$ is m-accretive. Under some assumptions, $\{x_{n}\}$ is proved to be convergent strongly to the unique element $p_{0}\in N(A)$, which solves the following variational inequality:

1.10 $$ \bigl\langle p_{0} - f(p_{0}), J(p_{0} - u) \bigr\rangle \leq0, \quad\forall u \in N(A). $$

The mapping *F* in (1.9) is called a perturbed operator which only plays a role in the construction of the iterative algorithm for selecting a particular zero of *A*, and it is not involved in the variational inequality (1.10).

Inspired by the work mentioned above, in Section 2, we shall construct a new modified forward-backward splitting midpoint iterative algorithm to approximate the zero points of the sum of infinite *m*-accretive mappings and infinite $\theta_{i}$-inversely strongly accretive mappings. New proof techniques can be found, the superposition of perturbed operators is considered and some restrictions on the parameters are mild compared to the existing similar works. In Section 3, we shall discuss the applications of the newly obtained iterative algorithms to integro-differential systems and the convex minimization problems.

We need the following preliminaries in our paper.

### Lemma 1.1

[12]

*Let* *X* *be a real uniformly convex and* *p*-*uniformly smooth Banach space with constant* $K_{p}$ *for some* $p \in(1,2]$. *Let* *D* *be a nonempty closed convex subset of* *X*. *Let* $A: D\rightarrow X$ *be an* *m*-*accretive mapping and* $B: D\rightarrow X$ *be a* *θ*-*inversely strongly accretive mapping*. *Then*, *given* $s > 0$, *there exists a continuous*, *strictly increasing and convex function* $\varphi_{p}: R^{+} \rightarrow R^{+}$ *with* $\varphi_{p}(0) = 0$ *such that for all* $x, y \in D$ *with* $\Vert x \Vert \leq s$ *and* $\Vert y \Vert \leq s$,

$$\begin{gathered} \bigl\Vert J_{r}^{A}(I-rB)x-J_{r}^{A}(I-rB)y \bigr\Vert ^{p} \\ \quad\leq \Vert x-y \Vert ^{p} - r \bigl(p \theta- K_{p} r^{p-1} \bigr) \Vert Bx-By \Vert ^{p}\\ \qquad {}- \varphi_{p} \bigl( \bigl\Vert \bigl(I-J_{r}^{A} \bigr) (I-rB)x- \bigl(I-J_{r}^{A} \bigr) (I-rB)y \bigr\Vert \bigr). \end{gathered} $$

*In particular*, *if* $0 < r \leq(\frac{p \theta}{K_{p}})^{\frac{1}{p-1}}$, *then* $J_{r}^{A}(I-rB)$ *is non*-*expansive*.

### Lemma 1.2

[13]

*Let* *X* *be a real smooth Banach space and* $B: X \rightarrow X$ *be a* *μ*-*strictly pseudo*-*contractive mapping and also be a* *γ*-*strongly accretive mapping with* $\mu+ \gamma> 1$. *Then*, *for any fixed number* $\delta\in(0,1)$, $I-\delta B$ *is a contraction with coefficient* $1-\delta(1-\sqrt{\frac{1-\gamma }{\mu}})$.

### Lemma 1.3

[2]

*Let* *X* *be a real Banach space and* *D* *be a nonempty closed and convex subset of* *X*. *Let* $f: D \rightarrow D$ *be a contraction*. *Then* *f* *has a unique fixed point*.

### Lemma 1.4

[14]

*Let* *X* *be a real strictly convex Banach space*, *and let* *D* *be a nonempty closed and convex subset of* *X*. *Let* $T_{m}: D \rightarrow D$ *be a non*-*expansive mapping for each* $m \in N$. *Let* $\{a_{m}\}$ *be a real number sequence in* (0,1) *such that* $\sum_{m = 1}^{\infty}a_{m} = 1$. *Suppose that* $\bigcap_{m=1}^{\infty}F(T_{m}) \neq\emptyset$. *Then the mapping* $\sum_{m = 1}^{\infty}a_{m} T_{m}$ *is non*-*expansive and* $F(\sum_{m = 1}^{\infty}a_{m}T_{m}) = \bigcap_{m = 1}^{\infty}F(T_{m})$.

### Lemma 1.5

[12]

*In a real Banach space* *X*, *for* $p > 1$, *the following inequality holds*:

$$\Vert x+y \Vert ^{p} \leq \Vert x \Vert ^{p} + p \bigl\langle y, j_{p}(x+y) \bigr\rangle , \quad\forall x,y \in X, j_{p}(x+y) \in J_{p}(x+y). $$

### Lemma 1.6

[15]

*Let* *X* *be a real Banach space*, *and let* *D* *be a nonempty closed and convex subset of* *X*. *Suppose* $A: D \rightarrow X$ *is a single*-*valued mapping and* $B: X \rightarrow 2^{X}$ *is m*-*accretive*. *Then*

$$F \bigl((I+rB)^{-1}(I-rA) \bigr) = N(A+B) \quad\textit{for }\forall r > 0. $$

### Lemma 1.7

[16]

*Let* $\{a_{n}\}$ *be a real sequence that does not decrease at infinity*, *in the sense that there exists a subsequence* $\{a_{n_{k}}\}$ *so that* $a_{n_{k}}\leq a_{n_{k}+1}$ *for all* $k \in N\cup\{0\}$. *For every* $n > n_{0}$, *define an integer sequence* $\{ \tau(n)\}$ *as*

$$\tau(n) = \max\{n_{0} \leq k \leq n: a_{k} < a_{k+1}\}. $$

*Then* $\tau(n) \rightarrow\infty$ *as* $n \rightarrow\infty$ *and for all* $n > n_{0}$, $\max\{a_{\tau(n)}, a_{n}\} \leq a_{\tau(n)+1}$.

### Lemma 1.8

[17]

*For* $p > 1$, *the following inequality holds*:

$$ab \leq\frac{1}{p} a^{p} + \frac{p-1}{p}b^{\frac{p}{p-1}}, $$

*for any positive real numbers* *a* *and* *b*.

### Lemma 1.9

[18]

*The Banach space* *X* *is uniformly smooth if and only if the duality mapping* $J_{p}$ *is single*-*valued and norm*-*to*-*norm uniformly continuous on bounded subsets of* *X*.

## Strong convergence theorems

### Theorem 2.1

*Let* *X* *be a real uniformly convex and* *p*-*uniformly smooth Banach space with constant* $K_{p}$ *where* $p \in(1,2]$ *and* *D* *be a nonempty closed and convex sunny non*-*expansive retract of* *X*. *Let* $Q_{D}$ *be the sunny non*-*expansive retraction of* *X* *onto* *D*. *Let* $f: X \rightarrow X$ *be a contraction with coefficient* $k \in(0,1)$, $A_{i}: D \rightarrow X$ *be m*-*accretive mappings*, $C_{i}: D \rightarrow X$ *be* $\theta_{i}$-*inversely strongly accretive mappings*, $W_{i}: X \rightarrow X$ *be* $\mu_{i}$-*strictly pseudo*-*contractive mappings and* $\gamma_{i}$-*strongly accretive mappings with* $\mu_{i}+\gamma_{i} > 1$ *for* $i\in N$. *Suppose* $\{\omega_{i}^{(1)}\}$ *and* $\{\omega_{i}^{(2)}\}$ *are real number sequences in* $(0,1)$ *for* $i \in N$. *Suppose* $0 < r_{n,i}\leq(\frac{p\theta_{i}}{K_{p}})^{\frac{1}{p-1}}$ *for* $i \in N$ *and* $n \in N$, $\kappa_{t} \in(0,1)$ *for* $t \in(0,1)$, $\sum_{i = 1}^{\infty }\omega_{i}^{(1)} \Vert W_{i} \Vert <+\infty$, $\sum_{i = 1}^{\infty} \omega_{i}^{(1)} = \sum_{i = 1}^{\infty} \omega_{i}^{(2)}= 1$ *and* $\bigcap_{i = 1}^{\infty }N(A_{i}+C_{i})\neq\emptyset$. *If*, *for each* $t \in(0,1)$, *we define* $Z_{t}^{n}: X \rightarrow X$ *by*

$$Z_{t}^{n} u = t f(u) + (1-t) \Biggl(I-\kappa_{t} \sum_{i = 1}^{\infty} \omega _{i}^{(1)}W_{i} \Biggr) \Biggl(\sum_{i = 1}^{\infty} \omega _{i}^{(2)}J_{r_{n,i}}^{A_{i}}(I-r_{n,i}C_{i})Q_{D} u \Biggr), $$

*then* $Z_{t}^{n}$ *has a fixed point* $u_{t}^{n}$. *Moreover*, *if* $\frac{\kappa _{t}}{t} \rightarrow0$, *then* $u_{t}^{n}$ *converges strongly to the unique solution* $q_{0}$ *of the following variational inequality*, *as* $t \rightarrow0$:

2.1 $$ \bigl\langle q_{0} - f(q_{0}), J(q_{0} - u) \bigr\rangle \leq0, \quad\forall u \in\bigcap_{i = 1}^{\infty}N(A_{i}+C_{i}). $$

### Proof

We split the proof into five steps.

Step 1. $Z_{t}^{n}: X \rightarrow X$ is a contraction for $t \in(0,1)$, $\kappa_{t} \in(0,1)$ and $n \in N$.

In fact, for $\forall x,y \in X$, using Lemmas 1.1 and 1.2, we have

$$\begin{gathered} \bigl\Vert Z_{t}^{n} x - Z_{t}^{n} y \bigr\Vert \\ \quad\leq t \bigl\Vert f(x) - f(y) \bigr\Vert + (1-t)\times \Biggl\Vert \sum _{i = 1}^{\infty} \omega_{i}^{(1)}(I- \kappa_{t}W_{i}) \Biggl(\sum_{i = 1}^{\infty} \omega_{i}^{(2)}J_{r_{n,i}}^{A_{i}}(I-r_{n,i}C_{i})Q_{D}x \Biggr) \\ \qquad{}-\sum_{i = 1}^{\infty} \omega_{i}^{(1)}(I-\kappa_{t} W_{i}) \Biggl(\sum_{i = 1}^{\infty} \omega_{i}^{(2)}J_{r_{n,i}}^{A_{i}}(I-r_{n,i}C_{i})Q_{D}y \Biggr)\Biggr\Vert \\ \quad\leq tk \Vert x - y \Vert + (1-t)\sum_{i = 1}^{\infty} \omega _{i}^{(1)} \biggl[1-\kappa_{t} \biggl(1- \sqrt{\frac{1-\gamma_{i}}{\mu_{i}}} \biggr) \biggr] \Vert x-y \Vert \\ \quad \leq \bigl[1-(1-k)t \bigr] \Vert x-y \Vert , \end{gathered} $$

which implies that $Z_{t}^{n}$ is a contraction. By Lemma 1.3, there exists $u_{t}^{n}$ such that $Z_{t}^{n}u_{t}^{n} = u_{t}^{n}$. That is, $u_{t}^{n} = t f(u_{t}^{n}) + (1-t)(I-\kappa_{t} \sum_{i = 1}^{\infty} \omega_{i}^{(1)}W_{i})(\sum_{i = 1}^{\infty} \omega _{i}^{(2)}J_{r_{n,i}}^{A_{i}}(I-r_{n,i}C_{i})Q_{D}u_{t}^{n})$.

Step 2. If $\lim_{t \rightarrow0}\frac{\kappa_{t}}{t} = 0$, then $\{ u_{t}^{n}\}$ is bounded for $n \in N$, $0 < t \leq\overline{a}$, where *a̅* is a sufficiently small positive number and $u_{t}^{n}$ is the same as that in Step 1.

For $\forall u \in\bigcap_{i = 1}^{\infty}N(A_{i}+C_{i})$, using Lemmas 1.1, 1.2 and 1.6, we know that

$$\begin{aligned} \bigl\Vert u_{t}^{n} - u \bigr\Vert &\leq t k \bigl\Vert u_{t}^{n} - u \bigr\Vert +t \bigl\Vert f(u) - u \bigr\Vert +(1-t)\kappa_{t} \sum _{i = 1}^{\infty} \omega_{i}^{(1)} \Vert W_{i}u \Vert \\ &\quad{}+ (1-t)\sum_{i = 1}^{\infty} \omega_{i}^{(1)}\Biggl\Vert (I-\kappa_{t} W_{i}) \Biggl[\sum_{i = 1}^{\infty} \omega _{i}^{(2)}J_{r_{n,i}}^{A_{i}}(I-r_{n,i}C_{i})Q_{D}u_{t}^{n} \\ &\quad{}-\sum_{i = 1}^{\infty}\omega _{i}^{(2)}J_{r_{n,i}}^{A_{i}}(I-r_{n,i}C_{i})Q_{D}u \Biggr]\Biggr\Vert \\ &\leq t k \bigl\Vert u_{t}^{n} - u \bigr\Vert +t \bigl\Vert f(u) - u \bigr\Vert +(1-t)\kappa_{t} \sum _{i = 1}^{\infty} \omega_{i}^{(1)} \Vert W_{i} \Vert \Vert u \Vert \\ &\quad{} + (1-t)\sum_{i = 1}^{\infty} \omega_{i}^{(1)} \biggl[1-\kappa _{t} \biggl(1- \sqrt{ \frac{1-\gamma_{i}}{\mu_{i}}} \biggr) \biggr] \bigl\Vert u_{t}^{n}-u \bigr\Vert \\ & \leq t \bigl\Vert f(u) - u \bigr\Vert +(1-t+tk) \bigl\Vert u_{t}^{n} - u \bigr\Vert +(1-t)\kappa_{t} \sum _{i = 1}^{\infty} \omega_{i}^{(1)} \Vert W_{i} \Vert \Vert u \Vert . \end{aligned} $$

Then

$$\bigl\Vert u_{t}^{n} - u \bigr\Vert \leq \frac{ \Vert f(u) - u \Vert +\frac{\kappa_{t}}{t}\sum_{i = 1}^{\infty} \omega_{i}^{(1)} \Vert W_{i} \Vert \Vert u \Vert }{1-k}. $$

Since $\lim_{t \rightarrow0}\frac{\kappa_{t}}{t} = 0$, then there exists a sufficiently small positive number *a̅* such that $0 < \frac {\kappa_{t}}{t}< 1$ for $0 < t \leq\overline{a}$. Thus $\{u_{t}^{n}\}$ is bounded for $n \in N$ and $0 < t \leq\overline{a}$.

Step 3. If $\lim_{t \rightarrow0}\frac{\kappa_{t}}{t} = 0$, then $u_{t}^{n} - \sum_{i = 1}^{\infty} \omega _{i}^{(2)}J_{r_{n,i}}^{A_{i}}(I-r_{n,i}C_{i})Q_{D}u_{t}^{n} \rightarrow0$, as $t \rightarrow0$, for $n \in N$.

Noticing Step 2, we have

$$\begin{aligned} &\Biggl\Vert u_{t}^{n} - \sum _{i = 1}^{\infty} \omega _{i}^{(2)}J_{r_{n,i}}^{A_{i}}(I-r_{n,i}C_{i})Q_{D}u_{t}^{n} \Biggr\Vert \\ &\quad \leq t \bigl\Vert f \bigl(u_{t}^{n} \bigr) \bigr\Vert +t\sum_{i = 1}^{\infty} \omega _{i}^{(2)} \bigl\Vert J_{r_{n,i}}^{A_{i}}(I-r_{n,i}C_{i})Q_{D}u_{t}^{n} \bigr\Vert \\ &\qquad{}+ (1-t)\kappa_{t}\sum_{i = 1}^{\infty} \omega_{i}^{(1)} \Biggl\Vert W_{i} \Biggl(\sum _{i = 1}^{\infty}\omega _{i}^{(2)}J_{r_{n,i}}^{A_{i}}(I-r_{n,i}C_{i})Q_{D}u_{t}^{n} \Biggr) \Biggr\Vert \\ & \quad \rightarrow0, \end{aligned} $$

as $t \rightarrow0$.

Step 4. If the variational inequality (2.1) has solutions, the solution must be unique.

Suppose $u_{0} \in\bigcap_{i = 1}^{\infty}N(A_{i}+C_{i})$ and $v_{0} \in \bigcap_{i = 1}^{\infty}N(A_{i}+C_{i})$ are two solutions of (2.1), then

2.2 $$ \bigl\langle u_{0} - f(u_{0}), J(u_{0}-v_{0}) \bigr\rangle \leq0, $$

and

2.3 $$ \bigl\langle v_{0} - f(v_{0}), J(v_{0}-u_{0}) \bigr\rangle \leq0. $$

Adding up (2.2) and (2.3), we get

2.4 $$ \bigl\langle u_{0} - f(u_{0}) - v_{0} + f(v_{0}), J(u_{0}-v_{0}) \bigr\rangle \leq0. $$

Since

$$\begin{gathered} \bigl\langle u_{0} - f(u_{0}) - v_{0} + f(v_{0}), J(u_{0}-v_{0}) \bigr\rangle \\ \quad = \Vert u_{0} - v_{0} \Vert ^{2} - \bigl\langle f(u_{0})-f(v_{0}), J(u_{0}-v_{0}) \bigr\rangle \\ \quad \geq \Vert u_{0} - v_{0} \Vert ^{2} - k \Vert u_{0} - v_{0} \Vert ^{2} = (1-k) \Vert u_{0} - v_{0} \Vert ^{2}, \end{gathered} $$

then (2.4) implies that $u_{0} = v_{0}$.

Step 5. If $\lim_{t \rightarrow0}\frac{\kappa_{t}}{t} = 0$, then $u_{t}^{n} \rightarrow q_{0} \in\bigcap_{i = 1}^{\infty}N(A_{i}+C_{i})$, as $t \rightarrow0$, which solves the variational inequality (2.1).

Assume $t_{m} \rightarrow0$. Set $u_{m}^{n}: = u_{t_{m}}^{n}$ and define $\mu: X \rightarrow R$ by

$$\mu(u) = \operatorname{LIM} \bigl\Vert u_{m}^{n} - u \bigr\Vert ^{2}, \quad u \in X, $$

where LIM is the Banach limit on $l^{\infty}$. Let

$$K = \Bigl\{ x \in X: \mu(x) = \min_{x \in X}\operatorname{LIM} \bigl\Vert u_{m}^{n} -x \bigr\Vert ^{2} \Bigr\} . $$

It is easily seen that *K* is a nonempty closed convex bounded subset of *X*. Since $u_{m}^{n} - \sum_{i = 1}^{\infty} \omega _{i}^{(2)}J_{r_{n,i}}^{A_{i}}(I-r_{n,i}C_{i})Q_{D}u_{m}^{n} \rightarrow0$ from Step 3, then for $u \in K$,

$$\begin{aligned} \mu \Biggl(\sum_{i = 1}^{\infty} \omega _{i}^{(2)}J_{r_{n,i}}^{A_{i}}(I-r_{n,i}C_{i})Q_{D}u \Biggr) &= \operatorname{LIM} \Biggl\Vert u_{m}^{n} - \sum _{i = 1}^{\infty} \omega _{i}^{(2)}J_{r_{n,i}}^{A_{i}}(I-r_{n,i}C_{i})Q_{D}u \Biggr\Vert ^{2}\\ &\leq \operatorname{LIM} \bigl\Vert u_{m}^{n} - u \bigr\Vert ^{2} = \mu(u), \end{aligned} $$

it follows that $\sum_{i = 1}^{\infty}\omega _{i}^{(2)}J_{r_{n,i}}^{A_{i}}(I-r_{n,i}C_{i})Q_{D}(K) \subset K$; that is, *K* is invariant under $\sum_{i = 1}^{\infty} \omega _{i}^{(2)}J_{r_{n,i}}^{A_{i}}(I-r_{n,i}C_{i})Q_{D}$. Since a uniformly smooth Banach space has the fixed point property for non-expansive mappings, $\sum_{i = 1}^{\infty} \omega _{i}^{(2)}J_{r_{n,i}}^{A_{i}}(I-r_{n,i}C_{i})Q_{D}$ has a fixed point, say $q_{0}$, in *K*. That is, $\sum_{i = 1}^{\infty} \omega _{i}^{(2)}J_{r_{n,i}}^{A_{i}}(I-r_{n,i}C_{i})Q_{D}q_{0} = q_{0} \in D$, which ensures from Lemmas 1.4 and 1.6 that $q_{0} \in\bigcap_{i = 1}^{\infty}N(A_{i}+C_{i})$. Since $q_{0}$ is also a minimizer of *μ* over *X*, it follows that, for $t \in(0,1)$,

$$\begin{aligned} 0 &\leq\frac{\mu(q_{0}+tf(q_{0})-tq_{0})-\mu(q_{0})}{t} \\ &= \operatorname{LIM}\frac{ \Vert u_{m}^{n}-q_{0}- t f(q_{0})+tq_{0} \Vert ^{2}- \Vert u_{m}^{n} - q_{0} \Vert ^{2}}{t} \\ &= \operatorname{LIM} \frac{ \langle u_{m}^{n}-q_{0}- tf(q_{0}) +t q_{0}, J(u_{m}^{n}-q_{0}- tf(q_{0}) +t q_{0})\rangle - \Vert u_{m}^{n} -q_{0} \Vert ^{2}}{t} \\ & = \operatorname{LIM} \bigl( \bigl\langle u_{m}^{n}-q_{0}, J \bigl(u_{m}^{n}-q_{0}- t f(q_{0}) +t q_{0} \bigr) \bigr\rangle \\ &\quad{} + t \bigl\langle q_{0}-f(q_{0}), J \bigl(u_{m}^{n}-q_{0}- t f(q_{0}) +tq_{0} \bigr) \bigr\rangle - \bigl\Vert u_{m}^{n} - q_{0} \bigr\Vert ^{2} \bigr)/t. \end{aligned} $$

Since *X* is uniformly smooth, then by letting $t \rightarrow0$, we find the two limits above can be interchanged and obtain

2.5 $$ \operatorname{LIM} \bigl\langle f(q_{0})-q_{0}, J \bigl(u_{m}^{n} - q_{0} \bigr) \bigr\rangle \leq0. $$

Since $u_{m}^{n} - q_{0} = t_{m}(f(u_{m}^{n})-q_{0})+(1-t_{m})[(I-\kappa_{t_{m}} \sum_{i = 1}^{\infty} \omega_{i}^{(1)}W_{i})(\sum_{i = 1}^{\infty}\omega _{i}^{(2)}J_{r_{n,i}}^{A_{i}}(I- r_{n,i}C_{i})Q_{D}u_{m}^{n})-q_{0}]$, then

$$\begin{aligned} \bigl\Vert u_{m}^{n} - q_{0} \bigr\Vert ^{2} &= \bigl\langle u_{m}^{n} - q_{0}, J \bigl(u_{m}^{n} - q_{0} \bigr) \bigr\rangle \\ &\leq t_{m} \bigl\langle f \bigl(u_{m}^{n} \bigr) - f(q_{0}), J \bigl(u_{m}^{n} - q_{0} \bigr) \bigr\rangle + t_{m} \bigl\langle f(q_{0})- q_{0}, J \bigl(u_{m}^{n}-q_{0} \bigr) \bigr\rangle \\ &\quad{}+ (1-t_{m}) \Biggl\Vert \sum_{i = 1}^{\infty} \omega _{i}^{(2)}J_{r_{n,i}}^{A_{i}}(I-r_{n,i}C_{i})Q_{D}u_{m}^{n} - q_{0} \Biggr\Vert \bigl\Vert u_{m}^{n}-q_{0} \bigr\Vert \\ &\quad{}+(1-t_{m})\kappa_{t_{m}} \Biggl\Vert \sum _{i = 1}^{\infty}\omega _{i}^{(1)}W_{i} \Biggl(\sum_{i = 1}^{\infty} \omega _{i}^{(2)}J_{r_{n,i}}^{A_{i}}(I-r_{n,i}C_{i})Q_{D}u_{m}^{n} \Biggr) \Biggr\Vert \bigl\Vert u_{m}^{n}-q_{0} \bigr\Vert \\ & \leq(1-t_{m}+t_{m}k) \bigl\Vert u_{m}^{n} -q_{0} \bigr\Vert ^{2}+t_{m} \bigl\langle f(q_{0})- q_{0}, J \bigl(u_{m}^{n}-q_{0} \bigr) \bigr\rangle \\ &\quad{}+(1-t_{m})\kappa_{t_{m}} \Biggl\Vert \sum _{i = 1}^{\infty}\omega _{i}^{(1)}W_{i} \Biggl(\sum_{i = 1}^{\infty} \omega _{i}^{(2)}J_{r_{n,i}}^{A_{i}}(I-r_{n,i}C_{i})Q_{D}u_{m}^{n} \Biggr) \Biggr\Vert \bigl\Vert u_{m}^{n}-q_{0} \bigr\Vert . \end{aligned} $$

Therefore,

2.6 $$ \begin{aligned}[b] \bigl\Vert u_{m}^{n} - q_{0} \bigr\Vert ^{2} &\leq \frac{1}{1-k} \Biggl[ \bigl\langle f(q_{0}) - q_{0}, J \bigl(u_{m}^{n}-q_{0} \bigr) \bigr\rangle \\ &\quad{}+ \frac{\kappa_{t_{m}}}{t_{m}} \Biggl\Vert \sum_{i = 1}^{\infty } \omega_{i}^{(1)}W_{i} \Biggl(\sum _{i = 1}^{\infty} \omega _{i}^{(2)}J_{r_{n,i}}^{A_{i}}(I-r_{n,i}C_{i})Q_{D}u_{m}^{n} \Biggr) \Biggr\Vert \bigl\Vert u_{m}^{n}-q_{0} \bigr\Vert \Biggr]. \end{aligned} $$

Since $\frac{\kappa_{t_{m}}}{t_{m}} \rightarrow0$, then from (2.5), (2.6) and the result of Step 2, we have $\operatorname{LIM} \Vert u_{m}^{n} - q_{0} \Vert ^{2} \leq0$, which implies that $\operatorname{LIM} \Vert u_{m}^{n} - q_{0} \Vert ^{2}=0$, and then there exists a subsequence which is still denoted by $\{u_{m}^{n}\}$ such that $u_{m}^{n} \rightarrow q_{0}$.

Next, we shall show that $q_{0}$ solves the variational inequality (2.1).

Note that $u_{m}^{n} = t_{m}f(u_{m}^{n})+(1-t_{m})(I-\kappa_{t_{m}}\sum_{i = 1}^{\infty}\omega_{i}^{(1)}W_{i})(\sum_{i = 1}^{\infty} \omega _{i}^{(2)}J_{r_{n,i}}^{A_{i}}(I-r_{n,i}C_{i})Q_{D}u_{m}^{n})$, then for $\forall v \in\bigcap_{i = 1}^{\infty}N(A_{i}+C_{i})$,

$$\begin{aligned}& \Biggl\langle \sum _{i = 1}^{\infty}\omega _{i}^{(2)}J_{r_{n,i}}^{A_{i}}(I-r_{n,i}C_{i})Q_{D}u_{m}^{n} - f \bigl(u_{m}^{n} \bigr),J \bigl(u_{m}^{n} - v \bigr) \Biggr\rangle \\& \quad = \frac{1}{t_{m}} \Biggl\langle \Biggl(I-\kappa_{t_{m}}\sum _{i = 1}^{\infty}\omega _{i}^{(1)}W_{i} \Biggr) \Biggl(\sum_{i = 1}^{\infty}\omega _{i}^{(2)}J_{r_{n,i}}^{A_{i}}(I-r_{n,i}C_{i})Q_{D}u_{m}^{n} \Biggr), J \bigl(u_{m}^{n} - v \bigr) \Biggr\rangle \\& \qquad{} -\frac{1}{t_{m}} \Biggl\langle u_{m}^{n} -t_{m}\kappa_{t_{m}}\sum_{i = 1}^{\infty} \omega_{i}^{(1)}W_{i} \Biggl(\sum _{i = 1}^{\infty} \omega _{i}^{(2)}J_{r_{n,i}}^{A_{i}}(I-r_{n,i}C_{i})Q_{D}u_{m}^{n} \Biggr), J \bigl(u_{m}^{n} - v \bigr) \Biggr\rangle \\& \begin{gathered} \quad = \frac{1}{t_{m}} \Biggl\langle \sum_{i = 1}^{\infty} \omega _{i}^{(1)}(I-\kappa_{t_{m}}W_{i}) \Biggl(\sum_{i = 1}^{\infty} \omega _{i}^{(2)}J_{r_{n,i}}^{A_{i}}(I-r_{n,i}C_{i})Q_{C}u_{m}^{n} \Biggr) \\ \qquad{}-\sum_{i = 1}^{\infty} \omega_{i}^{(1)}(I-\kappa_{t_{m}}W_{i}) \Biggl(\sum_{i = 1}^{\infty} \omega_{i}^{(2)}J_{r_{n,i}}^{A_{i}}(I-r_{n,i}C_{i})Q_{D}v \Biggr), J \bigl(u_{m}^{n} - v \bigr) \Biggr\rangle \\ \qquad{}-\frac{1}{t_{m}} \bigl\Vert u_{m}^{n}-v \bigr\Vert ^{2} - \frac{\kappa _{t_{m}}}{t_{m}} \Biggl\langle \sum _{i = 1}^{\infty}\omega_{i}^{(1)}W_{i} v, J \bigl(u_{m}^{n} - v \bigr) \Biggr\rangle \\ \qquad{}+\kappa_{t_{m}} \Biggl\langle \sum_{i = 1}^{\infty} \omega _{i}^{(1)}W_{i} \Biggl(\sum _{i = 1}^{\infty}\omega _{i}^{(2)}J_{r_{n,i}}^{A_{i}}(I-r_{n,i}C_{i})Q_{D}u_{m}^{n} \Biggr), J \bigl(u_{m}^{n} - v \bigr) \Biggr\rangle \end{gathered} \\& \quad \leq-\frac{1}{t_{m}} \Biggl\{ 1-\sum_{i = 1}^{\infty} \omega _{i}^{(1)} \biggl[1-\kappa_{t_{m}} \biggl(1- \sqrt{ \frac{1-\gamma_{i}}{\mu_{i}}} \biggr) \biggr] \Biggr\} \bigl\Vert u_{m}^{n} - v \bigr\Vert ^{2} \\& \qquad {} + \frac{\kappa_{t_{m}}}{t_{m}}\sum_{i = 1}^{\infty} \omega_{i}^{(1)} \Vert W_{i} \Vert \Vert v \Vert \bigl\Vert u_{m}^{n} - v \bigr\Vert \\& \qquad {}+\kappa_{t_{m}}\sum_{i = 1}^{\infty} \omega_{i}^{(1)} \Biggl\Vert W_{i} \Biggl(\sum _{i = 1}^{\infty} \omega _{i}^{(2)}J_{r_{n,i}}^{A_{i}}(I-r_{n,i}C_{i})Q_{D}u_{m}^{n} \Biggr) \Biggr\Vert \bigl\Vert u_{m}^{n} - v \bigr\Vert \\& \quad \leq\frac{\kappa_{t_{m}}}{t_{m}}\sum_{i = 1}^{\infty} \omega _{i}^{(1)} \Vert W_{i} \Vert \Vert v \Vert \bigl\Vert u_{m}^{n} - v \bigr\Vert \\& \qquad{} +\kappa_{t_{m}}\sum_{i = 1}^{\infty} \omega_{i}^{(1)} \Biggl\Vert W_{i} \Biggl(\sum _{i = 1}^{\infty} \omega _{i}^{(2)}J_{r_{n,i}}^{A_{i}}(I-r_{n,i}C_{i})Q_{D}u_{m}^{n} \Biggr) \Biggr\Vert \bigl\Vert u_{m}^{n} - v \bigr\Vert \\& \quad\rightarrow0, \end{aligned}$$

as $t_{m} \rightarrow0$. Since $x_{n} \rightarrow q_{0}$ and *J* is uniformly continuous on each bounded subset of *X*, then taking the limits on both sides of the above inequality, $\langle q_{0} - f(q_{0}), J(q_{0} - v)\rangle\leq0$, which implies that $q_{0}$ satisfies the variational inequality (2.1).

Next, to prove the net $\{u_{m}^{n}\}$ converges strongly to $q_{0}$, as $t \rightarrow0$, suppose that there is another subsequence $\{u_{t_{k}}^{n}\} $ of $\{u_{t}^{n}\}$ satisfying $u_{t_{k}}^{n} \rightarrow v_{0}$ as $t_{k} \rightarrow0$. Denote $u_{t_{k}}^{n} $ by $u_{k}^{n}$. Then the result of Step 3 implies that $0 = \lim_{t_{k} \rightarrow0}(u_{k}^{n} - \sum_{i = 1}^{\infty} \omega _{i}^{(2)}J_{r_{n,i}}^{A_{i}}(I-r_{n,i}C_{i})Q_{D}u_{k}^{n}) = v_{0} - \sum_{i = 1}^{\infty} \omega_{i}^{(2)}J_{r_{n,i}}^{A_{i}}(I-r_{n,i}C_{i})Q_{D}v_{0}$, which ensures that $v_{0}\in\bigcap_{i = 1}^{\infty}N(A_{i}+C_{i})$ in view of Lemmas 1.4 and 1.6. Repeating the above proof, we can also know that $v_{0}$ solves the variational inequality (2.1). Thus $q_{0} = v_{0}$ by using the result of Step 4.

Hence $u_{t}^{n} \rightarrow q_{0}$, as $t \rightarrow0$, which is the unique solution of the variational inequality (2.1).

This completes the proof. □

### Theorem 2.2

*Let* *X* *be a real uniformly convex and* *p*-*uniformly smooth Banach space with constant* $K_{p}$ *where* $p \in(1,2]$ *and* *D* *be a nonempty closed and convex sunny non*-*expansive retract of* *X*. *Let* $Q_{D}$ *be the sunny non*-*expansive retraction of* *X* *onto* *D*. *Let* $f: X \rightarrow X$ *be a contraction with coefficient* $k \in(0,1)$, $A_{i}: D \rightarrow X$ *be m*-*accretive mappings*, $C_{i}: D \rightarrow X$ *be* $\theta_{i}$-*inversely strongly accretive mappings*, *and* $W_{i}: X \rightarrow X$ *be* $\mu_{i}$-*strictly pseudo*-*contractive mappings and* $\gamma_{i}$-*strongly accretive mappings with* $\mu_{i}+\gamma_{i} > 1$ *for* $i\in N$. *Suppose* $\{\omega_{i}^{(1)}\}$, $\{\omega_{i}^{(2)}\}$, $\{\alpha_{n}\}$, $\{ \beta_{n}\}$, $\{\vartheta_{n}\}$, $\{\nu_{n}\}$, $\{\xi_{n}\}$, $\{\delta_{n}\}$ *and* $\{\zeta_{n}\}$ *are real number sequences in* $(0,1)$, $\{r_{n,i}\} \subset(0,+\infty)$, $\{a_{n}\}\subset X$ *and* $\{b_{n}\}\subset D$ *are error sequences*, *where* $n \in N$ *and* $i \in N$. *Suppose* $\bigcap_{i = 1}^{\infty}N(A_{i}+C_{i})\neq\emptyset$. *Let* $\{x_{n}\} $ *be generated by the following iterative algorithm*:

2.7 $$\begin{aligned} \textstyle\begin{cases} x_{1} \in D,\\ u_{n} = Q_{D}(\alpha_{n} x_{n}+\beta_{n} a_{n}),\\ v_{n} = \vartheta_{n} u_{n}+\nu_{n}\sum_{i = 1}^{\infty}\omega _{i}^{(2)}J^{A_{i}}_{r_{n,i}}(I-r_{n,i}C_{i})(\frac{u_{n}+v_{n}}{2})+\xi_{n} b_{n},\\ x_{n+1}= \delta_{n} f(x_{n})+(1-\delta_{n})(I-\zeta_{n}\sum_{i = 1}^{\infty }\omega_{i}^{(1)}W_{i})\sum_{i = 1}^{\infty}\omega _{i}^{(2)}J^{A_{i}}_{r_{n,i}}(I-r_{n,i}C_{i})(\frac{u_{n}+v_{n}}{2}), \quad n \in N.\hspace{-20pt} \end{cases}\displaystyle \end{aligned}$$

*Under the following assumptions*:

(i) $\alpha_{n}+\beta_{n} \leq1$, $\vartheta_{n} + \nu_{n}+\xi_{n} \equiv1$ *for* $n \in N$;
(ii) $\sum_{i = 1}^{\infty} \omega_{i}^{(1)} = \sum_{i = 1}^{\infty} \omega_{i}^{(2)}= 1$;
(iii) $\sum_{n = 1}^{\infty} \Vert a_{n} \Vert <+ \infty$, $\sum_{n = 1}^{\infty} \Vert b_{n} \Vert <+ \infty$, $\sum_{n = 1}^{\infty} (1-\alpha_{n}) <+ \infty$, $\sum_{n = 1}^{\infty} \xi_{n} <+ \infty$, $\lim_{n \rightarrow\infty} \sum_{i = 1}^{\infty}r_{n,i} = 0$;
(iv) $\lim_{n \rightarrow\infty}\delta_{n} = 0$, $\sum_{n = 1}^{\infty } \delta_{n} = + \infty$;
(v) $1-\alpha_{n}+ \Vert a_{n} \Vert = o(\delta_{n})$, $\xi_{n} = o(\delta_{n})$, $\zeta_{n} = o(\xi_{n})$, $\nu_{n} \nrightarrow 0$, *as* $n \rightarrow \infty$;
(vi) $\sum_{i = 1}^{\infty}\omega_{i}^{(1)} \Vert W_{i} \Vert <+ \infty$, $0 < r_{n,i}\leq(\frac{p\theta_{i}}{K_{p}})^{\frac{1}{p-1}}$ *for* $i \in N$, $n \in N$,

*the iterative sequence* $x_{n} \rightarrow q_{0}\in\bigcap_{i = 1}^{\infty }N(A_{i}+C_{i})$, *which is the unique solution of the variational inequality* (2.1).

### Proof

We split the proof into four steps.

Step 1. $\{v_{n}\}$ is well defined and so is $\{x_{n}\}$.

For $s, t\in(0,1)$, define $H_{s,t}: D \rightarrow D$ by $H_{s,t} x: = su+tH(\frac{u+x}{2}) + (1-s-t)v$, where $H: D \rightarrow D$ is non-expansive for $x \in D$ and $u,v \in D$. Then, for $\forall x,y \in D$,

$$\Vert H_{s,t} x - H_{s,t}y \Vert \leq t \biggl\Vert \frac{u+x}{2}-\frac{u+y}{2} \biggr\Vert \leq\frac{t}{2} \Vert x-y \Vert . $$

Thus $H_{s,t}$ is a contraction, which ensures from Lemma 1.3 that there exists $x_{s,t}\in D$ such that $H_{s,t} x_{s,t} = x_{s,t}$. That is, $x_{s,t} = su+tH(\frac{u+x_{s,t}}{2}) + (1-s-t)v$.

Since $\sum_{i = 1}^{\infty} \omega_{i}^{(2)} = 1$ and $J^{A_{i}}_{r_{n,i}}(I-r_{n,i}C_{i})$ is non-expansive for $n \in N$ and $i \in N$, then $\{v_{n}\}$ is well defined, which implies that $\{x_{n}\}$ is well defined.

Step 2. $\{x_{n}\}$ is bounded.

For $\forall p \in\bigcap_{i = 1}^{\infty}N(A_{i}+C_{i})$, we can easily know that

$$\Vert u_{n} - p \Vert \leq\alpha_{n} \Vert x_{n}-p \Vert +\beta_{n} \Vert a_{n} \Vert +(1-\alpha_{n}) \Vert p \Vert . $$

And

$$\begin{aligned} \Vert v_{n} - p \Vert &\leq \vartheta_{n} \Vert u_{n}-p \Vert +\nu_{n} \biggl\Vert \frac{u_{n}+v_{n}}{2}-p \biggr\Vert +\xi_{n} \Vert b_{n}-p \Vert \\ & \leq \biggl(\vartheta_{n}+\frac{\nu_{n}}{2} \biggr) \Vert u_{n}-p \Vert +\frac {\nu_{n}}{2} \Vert v_{n}-p \Vert + \xi_{n} \Vert b_{n}-p \Vert . \end{aligned} $$

Thus

2.8 $$ \begin{aligned}[b] \Vert v_{n} - p \Vert &\leq \biggl(\frac {2\vartheta_{n}+\nu_{n}}{2-\nu_{n}} \biggr) \Vert u_{n}-p \Vert + \frac{2\xi _{n}}{2-\nu_{n}} \Vert b_{n}-p \Vert \\ &\leq\alpha_{n} \Vert x_{n}-p \Vert +\beta_{n} \Vert a_{n} \Vert +(1-\alpha_{n}) \Vert p \Vert +2 \Vert b_{n} \Vert +\frac{2\xi_{n}}{2-\nu_{n}} \Vert p \Vert . \end{aligned} $$

Using Lemma 1.2 and (2.8), we have, for $n \in N$,

2.9 $$ \begin{aligned}[b] \Vert x_{n+1} - p \Vert &\leq \delta _{n} \bigl\Vert f(x_{n}) - f(p) \bigr\Vert +\delta_{n} \bigl\Vert f(p) - p \bigr\Vert \\ &\quad{}+ (1-\delta_{n}) \Biggl\Vert \Biggl(I-\zeta_{n} \sum_{i = 1}^{\infty }\omega_{i}^{(1)}W_{i} \Biggr)\sum_{i = 1}^{\infty}\omega _{i}^{(2)}J^{A_{i}}_{r_{n,i}}(I-r_{n,i}C_{i}) \biggl(\frac{u_{n}+v_{n}}{2} \biggr) - p \Biggr\Vert \hspace{-20pt} \\ &\leq\delta_{n}k \Vert x_{n}-p \Vert + \delta_{n} \bigl\Vert f(p) - p \bigr\Vert + (1-\delta_{n}) \zeta_{n}\sum_{i = 1}^{\infty}\omega _{i}^{(1)} \Vert W_{i} \Vert \Vert p \Vert \\ &\quad{}+ (1-\delta_{n})\Biggl\Vert \sum_{i = 1}^{\infty} \omega _{i}^{(1)}(I-\zeta_{n}W_{i})\sum _{i = 1}^{\infty}\omega _{i}^{(2)}J^{A_{i}}_{r_{n,i}}(I-r_{n,i}C_{i}) \biggl(\frac{u_{n}+v_{n}}{2} \biggr) \\ &\quad{} - \sum_{i = 1}^{\infty} \omega_{i}^{(1)}(I-\zeta_{n}W_{i})\sum _{i = 1}^{\infty}\omega_{i}^{(2)}J^{A_{i}}_{r_{n,i}}(I-r_{n,i}C_{i})p \Biggr\Vert \\ & \leq\delta_{n}k \Vert x_{n}-p \Vert + \delta_{n} \bigl\Vert f(p) - p \bigr\Vert + (1-\delta_{n}) \zeta_{n}\sum_{i = 1}^{\infty}\omega _{i}^{(1)} \Vert W_{i} \Vert \Vert p \Vert \\ &\quad{}+ (1-\delta_{n}) \Biggl[1-\zeta_{n} \Biggl(1-\sum _{i = 1}^{\infty}\omega _{i}^{(1)} \sqrt{\frac{1-\gamma_{i}}{\mu_{i}}} \Biggr) \Biggr]\\ &\quad {}\times \biggl[\alpha_{n} \Vert x_{n} - p \Vert +\beta_{n} \Vert a_{n} \Vert +(1-\alpha_{n}) \Vert p \Vert +2 \Vert b_{n} \Vert + \frac{\xi_{n}}{2-\nu_{n}} \Vert p \Vert \biggr] \\ & \leq \Biggl\{ (1-\delta_{n}) \Biggl[1-\zeta_{n} \Biggl(1- \sum_{i = 1}^{\infty}\omega _{i}^{(1)} \sqrt{\frac{1-\gamma_{i}}{\mu_{i}}} \Biggr) \Biggr] +\delta_{n}k \Biggr\} \Vert x_{n}-p \Vert + \delta_{n} \bigl\Vert f(p) - p \bigr\Vert \hspace{-20pt} \\ &\quad{}+ (1-\delta_{n}) \Biggl[1-\zeta_{n} \Biggl(1-\sum _{i = 1}^{\infty}\omega _{i}^{(1)} \sqrt{\frac{1-\gamma_{i}}{\mu_{i}}} \Biggr) \Biggr]\\ &\quad {}\times \biggl[\beta_{n} \Vert a_{n} \Vert +(1-\alpha_{n}) \Vert p \Vert + \Vert b_{n} \Vert +\frac{\xi_{n}}{2-\nu_{n}} \Vert p \Vert \biggr] \\ &\quad{}+ (1-\delta_{n})\zeta_{n}\sum _{i = 1}^{\infty}\omega_{i}^{(1)} \Vert W_{i} \Vert \Vert p \Vert . \end{aligned} $$

By using the inductive method, we can easily get the following result from (2.9):

$$\begin{aligned} \Vert x_{n+1}-p \Vert &\leq \max \biggl\{ \Vert x_{1} - p \Vert , \frac{\sum_{i = 1}^{\infty}\omega_{i}^{(1)} \Vert W_{i} \Vert \Vert p \Vert }{1-\sum_{i = 1}^{\infty}\omega _{i}^{(1)}\sqrt{\frac{1-\gamma_{i}}{\mu_{i}}}}, \frac{ \Vert f(p)-p \Vert }{1-k} \biggr\} \\ &\quad{}+\sum_{k = 1}^{n}(1- \delta_{k}) \Biggl[1- \zeta_{k} \Biggl(1-\sum _{i = 1}^{\infty} \omega_{i}^{(1)}\sqrt{ \frac{1-\gamma_{i}}{\mu_{i}}} \Biggr) \Biggr]\\ &\quad {}\times \biggl[ \beta_{k} \Vert a_{k} \Vert +(1-\alpha_{k}) \Vert p \Vert + \Vert b_{k} \Vert +\frac{\xi_{k}}{2-\nu_{k}} \Vert p \Vert \biggr]. \end{aligned} $$

Therefore, from assumptions (iii) and (vi), we know that $\{x_{n}\}$ is bounded.

Step 3. There exists $q_{0} \in\bigcap_{i = 1}^{\infty}N(A_{i}+C_{i})$, which solves the variational inequality (2.1).

Using Theorem 2.1, we know that there exists $u_{t}^{n}$ such that $u_{t}^{n} = tf(u_{t}^{n})+(1-t)(I-\kappa_{t}\sum_{i = 1 }^{\infty}\omega_{i}^{(1)}W_{i})(\sum_{i = 1}^{\infty}\omega _{i}^{(2)}J_{r_{n,i}}^{A_{i}}(I-r_{n,i}C_{i})Q_{D}u_{t}^{n})$ for $t \in(0,1)$. Moreover, under the assumption that $\frac{\kappa_{t}}{t}\rightarrow0$, $u_{t}^{n} \rightarrow q_{0} \in\bigcap_{i = 1}^{\infty}N(A_{i}+C_{i})$, as $t \rightarrow0$, which is the unique solution of the variational inequality (2.1).

Step 4. $x_{n} \rightarrow q_{0}$, as $n \rightarrow\infty$, where $q_{0}$ is the same as that in Step 3.

Set $C_{1}:= \operatorname{sup}\{2 \Vert \alpha_{n}x_{n}+\beta_{n}a_{n}-q_{0} \Vert ^{p-1}, 2 \Vert q_{0} \Vert \Vert \alpha_{n}x_{n}+\beta _{n}a_{n}-q_{0} \Vert ^{p-1}: n \in N\}$, then from Step 2 and assumption (iii), $C_{1}$ is a positive constant. Using Lemma 1.5, we have

2.10 $$ \begin{aligned}[b] \Vert u_{n} - q_{0} \Vert ^{p} &\leq \alpha_{n} \Vert x_{n}-q_{0} \Vert ^{p}-p(1- \alpha_{n}) \bigl\langle q_{0}, J_{p}( \alpha_{n}x_{n}+\beta_{n}a_{n}-q_{0}) \bigr\rangle \\ &\quad{}+p\beta_{n} \bigl\langle a_{n}, J_{p}( \alpha_{n}x_{n}+\beta_{n}a_{n}-q_{0}) \bigr\rangle \\ &\leq \alpha_{n} \Vert x_{n}-q_{0} \Vert ^{p}+C_{1}(1-\alpha_{n})+C_{1} \Vert a_{n} \Vert . \end{aligned} $$

Using Lemma 1.1, we know that

$$\begin{aligned} \Vert v_{n}-q_{0} \Vert ^{p} &\leq\vartheta_{n} \Vert u_{n} - q_{0} \Vert ^{p}+\nu_{n} \sum _{i = 1}^{\infty} \omega_{i}^{(2)} \biggl\Vert J_{r_{n,i}}^{A_{i}}(I-r_{n,i}C_{i}) \biggl( \frac{u_{n}+v_{n}}{2} \biggr)-q_{0} \biggr\Vert ^{p} \\ &\quad {}+ \xi_{n} \Vert b_{n}-q_{0} \Vert ^{p} \\ &\leq \biggl(\vartheta_{n}+\frac{\nu_{n}}{2} \biggr) \Vert u_{n} - q_{0} \Vert ^{p}+\frac{\nu_{n}}{2} \Vert v_{n} - q_{0} \Vert ^{p} \\ &\quad {}- \nu_{n} \sum_{i = 1}^{\infty} \omega_{i}^{(2)}r_{n,i} \bigl(\theta_{i}p - r_{n,i}^{p-1}K_{p} \bigr) \biggl\Vert C_{i} \biggl(\frac{u_{n}+v_{n}}{2} \biggr)-C_{i}q_{0} \biggr\Vert ^{p} \\ &\quad{}-\nu_{n} \sum_{i = 1}^{\infty} \omega_{i}^{(2)}\varphi_{p} \biggl(\biggl\Vert \bigl(I-J^{A_{i}}_{r_{n,i}} \bigr) \biggl(\frac{u_{n}+v_{n}}{2}-r_{n,i}C_{i} \biggl(\frac {u_{n}+v_{n}}{2} \biggr) \biggr)\\ &\quad{}- \bigl(I-J^{A_{i}}_{r_{n,i}} \bigr) (q_{0} -r_{n,i}C_{i}q_{0}) \biggr\Vert \biggr) + \xi_{n} \Vert b_{n}-q_{0} \Vert^{p}. \end{aligned} $$

Therefore,

2.11 $$ \begin{aligned}[b] \Vert v_{n}-q_{0} \Vert ^{p} &\leq\frac {2\vartheta_{n}+\nu_{n}}{2-\nu_{n}} \Vert u_{n} - q_{0} \Vert ^{p}+ \frac {2\xi_{n}}{2-\nu_{n}} \Vert b_{n}-q_{0} \Vert ^{p} \\ &\quad{} -\frac{2\nu_{n}}{2-\nu_{n}} \sum_{i = 1}^{\infty} \omega _{i}^{(2)}r_{n,i} \bigl(\theta_{i}p - r_{n,i}^{p-1}K_{p} \bigr) \biggl\Vert C_{i} \biggl(\frac{u_{n}+v_{n}}{2} \biggr)-C_{i}q_{0} \biggr\Vert ^{p} \\ &\quad{}-\frac{2\nu_{n}}{2-\nu_{n}} \sum_{i = 1}^{\infty} \omega _{i}^{(2)}\varphi_{p} \biggl(\biggl\Vert \bigl(I-J^{A_{i}}_{r_{n,i}} \bigr) \biggl(\frac {u_{n}+v_{n}}{2}-r_{n,i}C_{i} \biggl(\frac{u_{n}+v_{n}}{2} \biggr) \biggr)\\ &\quad {}- \bigl(I-J^{A_{i}}_{r_{n,i}} \bigr) (q_{0} -r_{n,i}C_{i}q_{0})\biggr\Vert \biggr). \end{aligned} $$

Now, from (2.10)–(2.11) and Lemmas 1.4 and 1.5, we know that for $n\in N$,

$$\begin{aligned} & \Vert x_{n+1} - q_{0} \Vert ^{p}\\ &\quad = \Biggl\Vert \delta_{n} \bigl(f(x_{n}) - q_{0} \bigr)+ (1-\delta_{n}) \Biggl(\sum _{k = 1}^{\infty} \omega _{i}^{(2)}J_{r_{n,i}}^{A_{i}}(I-r_{n,i}C_{i}) \biggl(\frac{u_{n}+v_{n}}{2} \biggr) -q_{0} \Biggr) \\ &\qquad{} -(1-\delta_{n})\zeta_{n}\sum _{i = 1}^{\infty} \omega _{i}^{(1)}W_{i} \Biggl(\sum_{i = 1}^{\infty} \omega _{i}^{(2)}J_{r_{n,i}}^{A_{i}}(I-r_{n,i}C_{i}) \biggl(\frac{u_{n}+v_{n}}{2} \biggr) \Biggr)\Biggr\Vert ^{p} \\ &\quad \leq(1-\delta_{n}) \biggl\Vert \frac{u_{n}+v_{n}}{2}- q_{0} \biggr\Vert ^{p}\\ &\qquad {}+ p\delta_{n} \bigl\langle f(x_{n})-f(q_{0}), J_{p}(x_{n+1}-q_{0}) \bigr\rangle +p\delta _{n} \bigl\langle f(q_{0})-q_{0}, J_{p}(x_{n+1}-q_{0}) \bigr\rangle \\ &\qquad{} - p (1-\delta_{n})\zeta_{n} \Biggl\langle \sum _{i = 1}^{\infty}\omega _{i}^{(1)}W_{i} \Biggl(\sum_{i = 1}^{\infty}\omega _{i}^{(2)}J_{r_{n,i}}^{A_{i}}(I-r_{n,i}C_{i}) \biggl(\frac{u_{n}+v_{n}}{2} \biggr) \Biggr), J_{p}(x_{n+1}-q_{0}) \Biggr\rangle \\ &\quad \leq(1-\delta_{n}) \biggl(\frac{ \Vert u_{n} - p_{0} \Vert ^{2}}{2}+\frac { \Vert v_{n} - p_{0} \Vert ^{2}}{2} \biggr)\\ &\qquad {}+ p\delta_{n}k \Vert x_{n}-q_{0} \Vert \Vert x_{n+1}-q_{0} \Vert ^{p-1}+p\delta _{n} \bigl\langle f(q_{0})-q_{0}, J_{p}(x_{n+1}-q_{0}) \bigr\rangle \\ &\qquad{} - p (1-\delta_{n})\zeta_{n} \Biggl\langle \sum _{i = 1}^{\infty}\omega _{i}^{(1)}W_{i} \Biggl(\sum_{i = 1}^{\infty}\omega _{i}^{(2)}J_{r_{n,i}}^{A_{i}}(I-r_{n,i}C_{i}) \biggl(\frac{u_{n}+v_{n}}{2} \biggr) \Biggr), J_{p}(x_{n+1}-q_{0}) \Biggr\rangle \\ & \quad \leq(1-\delta_{n}) \Vert u_{n} - p_{0} \Vert ^{2}+(1-\delta_{n}) \Biggl[\frac {\xi_{n}}{2-\nu_{n}} \Vert b_{n}-q_{0} \Vert ^{p} \\ &\qquad{}-\frac{\nu_{n}}{2-\nu_{n}} \sum_{i = 1}^{\infty} \omega _{i}^{(2)}r_{n,i} \bigl(\theta_{i}p - r_{n,i}^{p-1}K_{p} \bigr) \biggl\Vert C_{i} \biggl(\frac{u_{n}+v_{n}}{2} \biggr)-C_{i}q_{0} \biggr\Vert ^{p} \\ &\qquad{}-\frac{\nu_{n}}{2-\nu_{n}} \sum_{i = 1}^{\infty} \omega _{i}^{(2)}\varphi_{p} \biggl(\biggl\Vert \bigl(I-J^{A_{i}}_{r_{n,i}} \bigr) \biggl(\frac {u_{n}+v_{n}}{2}-r_{n,i}C_{i} \biggl(\frac{u_{n}+v_{n}}{2} \biggr) \biggr)\\ &\qquad {}- \bigl(I-J^{A_{i}}_{r_{n,i}} \bigr) (q_{0} -r_{n,i}C_{i}q_{0})\biggr\Vert \biggr) \Biggr] \\ &\qquad{}+pk\delta_{n} \Vert x_{n}-q_{0} \Vert \Vert x_{n+1}-q_{0} \Vert ^{p-1}+p \delta_{n} \bigl\langle f(q_{0})-q_{0}, J_{p}(x_{n+1}-q_{0}) \bigr\rangle \\ &\qquad{}-p (1-\delta_{n})\zeta_{n} \Biggl\langle \sum _{i = 1}^{\infty}\omega _{i}^{(1)}W_{i} \Biggl(\sum_{i = 1}^{\infty}\omega _{i}^{(2)}J_{r_{n,i}}^{A_{i}}(I-r_{n,i}C_{i}) \biggl(\frac {u_{n}+v_{n}}{2} \biggr) \Biggr),J_{p}(x_{n+1}-q_{0}) \Biggr\rangle \\ &\quad{} \leq(1-\delta_{n}) \Vert x_{n} - p_{0} \Vert ^{p}+ C_{1} \bigl(1-\alpha_{n}+ \Vert a_{n} \Vert \bigr)+k\delta_{n} \Vert x_{n}-q_{0} \Vert ^{p}\\ &\qquad {}+k\delta_{n} \Vert x_{n+1}-q_{0} \Vert ^{p}+\frac{\xi_{n}}{2-\nu_{n}} \Vert b_{n}-q_{0} \Vert ^{p} \\ &\qquad{} -(1-\delta_{n})\frac{\nu_{n}}{2-\nu_{n}} \sum _{i = 1}^{\infty } \omega_{i}^{(2)} \varphi_{p} \biggl(\biggl\Vert \bigl(I-J^{A_{i}}_{r_{n,i}} \bigr) \biggl(\frac {u_{n}+v_{n}}{2}-r_{n,i}C_{i} \biggl( \frac{u_{n}+v_{n}}{2} \biggr) \biggr)\\ &\qquad {}- \bigl(I-J^{A_{i}}_{r_{n,i}} \bigr) (q_{0} -r_{n,i}C_{i}q_{0})\biggr\Vert \biggr) +p\delta_{n} \bigl\langle f(q_{0})-q_{0}, J_{p}(x_{n+1}-q_{0}) \bigr\rangle \\ &\qquad{}+2\zeta_{n}\sum_{i = 1}^{\infty} \omega_{i}^{(1)} \Biggl\Vert W_{i} \Biggl( \sum _{i = 1}^{\infty}\omega _{i}^{(2)}J_{r_{n,i}}^{A_{i}}(I-r_{n,i}C_{i}) \biggl(\frac{u_{n}+v_{n}}{2} \biggr) \Biggr) \Biggr\Vert \bigl\Vert J_{p}(x_{n+1}-q_{0}) \bigr\Vert , \end{aligned}$$

which implies that

$$\begin{aligned} &\Vert x_{n+1} - q_{0} \Vert ^{p}\\ &\quad \leq\frac{1-\delta _{n}(1-k)}{1-\delta_{n}k} \Vert x_{n} - q_{0} \Vert ^{p} + \frac {C_{1}(1-\alpha_{n}+ \Vert a_{n} \Vert )}{1-\delta_{n}k} \\ &\qquad{} + \frac{1}{1-\delta_{n}k} \Biggl(\frac{\xi_{n}}{2-\nu_{n}} \Vert b_{n}-q_{0} \Vert ^{p}+p\delta_{n} \bigl\langle f(q_{0})-q_{0}, J_{p}(x_{n+1}-q_{0}) \bigr\rangle \\ &\qquad{} +2\zeta_{n}\sum_{i = 1}^{\infty} \omega_{i}^{(1)} \Biggl\Vert W_{i} \Biggl( \sum _{i = 1}^{\infty}\omega _{i}^{(2)}J_{r_{n,i}}^{A_{i}}(I-r_{n,i}C_{i}) \biggl(\frac{u_{n}+v_{n}}{2} \biggr) \Biggr) \Biggr\Vert \bigl\Vert J_{p}(x_{n+1}-q_{0}) \bigr\Vert \Biggr) \\ &\qquad{} - (1-\delta_{n})\frac{\nu_{n}}{2-\nu_{n}}\frac{1}{1-\delta_{n}k}\sum _{i = 1}^{\infty}\omega_{i}^{(2)} \varphi_{p} \biggl(\biggl\Vert \bigl(I-J^{A_{i}}_{r_{n,i}} \bigr) \biggl(\frac{u_{n}+v_{n}}{2}-r_{n,i}C_{i} \biggl( \frac {u_{n}+v_{n}}{2} \biggr) \biggr)\\ &\qquad {}- \bigl(I-J^{A_{i}}_{r_{n,i}} \bigr) (q_{0} -r_{n,i}C_{i}q_{0})\biggr\Vert \biggr). \end{aligned}$$

From Step 2, if we set $C_{2} = \operatorname{sup} \{\sum_{i = 1}^{\infty}\omega _{i}^{(1)} \Vert W_{i}(\sum_{i = 1}^{\infty}\omega _{i}^{(2)}J_{r_{n,i}}^{A_{i}}(I-r_{n,i}C_{i})(\frac{u_{n}+v_{n}}{2})) \Vert , \Vert x_{n}-q_{0} \Vert ^{p-1}: n \in N\}$, then $C_{2}$ is a positive constant.

Let $\varepsilon_{n}^{(1)} = \frac{\delta_{n}(1-2k)}{1-\delta_{n}k}$, $\varepsilon_{n}^{(2)} = \frac{1}{\delta_{n}(1-2k)}[C_{1}(1-\alpha_{n}+ \Vert a_{n} \Vert )+\frac{\xi_{n}}{2-\nu_{n}} \Vert b_{n} - q_{0} \Vert ^{p} +p\delta_{n}\langle f(q_{0})-q_{0}, J_{p}(x_{n+1}-q_{0})\rangle+2\zeta _{n}C_{2}^{2}]$ and $\varepsilon_{n}^{(3)} = (1-\delta_{n})\frac{\nu_{n}}{2-\nu_{n}}\frac {1}{1-\delta_{n}k}\sum_{i = 1}^{\infty} \omega_{i}^{(2)}\varphi_{p}(\Vert (I-J^{A_{i}}_{r_{n,i}})(\frac{u_{n}+v_{n}}{2}-r_{n,i}C_{i}(\frac {u_{n}+v_{n}}{2}))-(I-J^{A_{i}}_{r_{n,i}})(q_{0} -r_{n,i}C_{i}q_{0})\Vert)$.

Then

2.12 $$ \Vert x_{n+1} - q_{0} \Vert ^{p} \leq \bigl(1 - \varepsilon _{n}^{(1)} \bigr) \Vert x_{n} - q_{0} \Vert ^{p} + \varepsilon _{n}^{(1)} \varepsilon_{n}^{(2)}- \varepsilon_{n}^{(3)}. $$

Our next discussion will be divided into two cases.

Case 1. $\{ \Vert x_{n} - q_{0} \Vert \}$ is decreasing.

If $\{ \Vert x_{n} - q_{0} \Vert \}$ is decreasing, we know from (2.12) and assumptions (iv) and (v) that

$$0 \leq\varepsilon_{n}^{(3)}\leq\varepsilon_{n}^{(1)} \bigl(\varepsilon _{n}^{(2)}- \Vert x_{n} - q_{0} \Vert ^{p} \bigr)+ \bigl( \Vert x_{n} - q_{0} \Vert ^{p}- \Vert x_{n+1} - q_{0} \Vert ^{p} \bigr)\rightarrow0, $$

which ensures that $\sum_{i = 1}^{\infty} \omega_{i}^{(2)}\varphi_{p}(\Vert (I-J^{A_{i}}_{r_{n,i}})(\frac{u_{n}+v_{n}}{2}-r_{n,i}C_{i}(\frac {u_{n}+v_{n}}{2}))-(I-J^{A_{i}}_{r_{n,i}})(q_{0} -r_{n,i}C_{i}q_{0})\Vert)\rightarrow0$, as $n \rightarrow+\infty$. Then, from the property of $\varphi_{p}$, we know that $\sum_{i = 1}^{\infty} \omega_{i}^{(2)}\Vert(I-J^{A_{i}}_{r_{n,i}})(\frac {u_{n}+v_{n}}{2}-r_{n,i}C_{i}(\frac{u_{n}+v_{n}}{2}))-(I-J^{A_{i}}_{r_{n,i}})(q_{0} -r_{n,i}C_{i}q_{0})\Vert\rightarrow0$, as $n \rightarrow+\infty$.

Note that $\lim_{n \rightarrow\infty}\sum_{i= 1}^{\infty}r_{n,i} = 0$, then

$$\begin{gathered} \Biggl\Vert \frac{u_{n}+v_{n}}{2}- \sum _{i = 1}^{\infty} \omega _{i}^{(2)}J^{A_{i}}_{r_{n,i}}(I-r_{n,i}C_{i}) \biggl(\frac{u_{n}+v_{n}}{2} \biggr) \Biggr\Vert \\ \quad\leq\sum_{i = 1}^{\infty} \omega_{i}^{(2)} \biggl\Vert \bigl(I-J^{A_{i}}_{r_{n,i}} \bigr) (I-r_{n,i}C_{i}) \biggl(\frac {u_{n}+v_{n}}{2} \biggr)- \bigl(I-J^{A_{i}}_{r_{n,i}} \bigr) (I-r_{n,i}C_{i})q_{0} \biggr\Vert \\ \qquad{}+ \sum_{i = 1}^{\infty} \omega_{i}^{(2)}r_{n,i} \biggl\Vert C_{i} \biggl(\frac{u_{n}+v_{n}}{2} \biggr) \biggr\Vert + \sum _{i = 1}^{\infty}\omega _{i}^{(2)}r_{n,i} \Vert C_{i}q_{0} \Vert \\ \quad \rightarrow0, \end{gathered} $$

as $n \rightarrow\infty$.

Now, our purpose is to show that $\operatorname{limsup}_{n \rightarrow\infty }\varepsilon_{n}^{(2)}\leq0$, which reduces to showing that $\operatorname{limsup}_{n \rightarrow\infty}\langle f(q_{0}) - q_{0}, J_{p}(x_{n+1}-q_{0})\rangle\leq0$.

Let $u_{t}^{n}$ be the same as that in Step 3. Since $\Vert u_{t}^{n} \Vert \leq \Vert u_{t}^{n} - q_{0} \Vert + \Vert q_{0} \Vert $, then $\{u_{t}^{n}\}$ is bounded, as $t \rightarrow0$. Using Lemma 1.5 again, we have

$$\begin{gathered} \biggl\Vert u_{t}^{n} - \frac{u_{n}+v_{n}}{2} \biggr\Vert ^{p} \\ \quad = \Biggl\Vert u_{t}^{n} - \sum _{i = 1}^{\infty} \omega _{i}^{(2)}J^{A_{i}}_{r_{n,i}}(I-r_{n,i}C_{i}) \biggl(\frac{u_{n}+v_{n}}{2} \biggr)\\ \qquad {}+\sum_{i = 1}^{\infty} \omega_{i}^{(2)}J^{A_{i}}_{r_{n,i}}(I-r_{n,i}C_{i}) \biggl(\frac {u_{n}+v_{n}}{2} \biggr) - \frac{u_{n}+v_{n}}{2} \Biggr\Vert ^{p} \\ \quad \leq \Biggl\Vert u_{t}^{n} - \sum _{i = 1}^{\infty} \omega _{i}^{(2)}J^{A_{i}}_{r_{n,i}}(I-r_{n,i}C_{i}) \biggl(\frac{u_{n}+v_{n}}{2} \biggr) \Biggr\Vert ^{p} \\ \qquad{} + p \Biggl\langle \sum_{i = 1}^{\infty} \omega _{i}^{(2)}J^{A_{i}}_{r_{n,i}}(I-r_{n,i}C_{i}) \biggl(\frac{u_{n}+v_{n}}{2} \biggr) - \frac {u_{n}+v_{n}}{2}, J_{p} \biggl(u_{t}^{n} - \frac{u_{n}+v_{n}}{2} \biggr) \Biggr\rangle \\ \quad = \Biggl\Vert tf \bigl(u_{t}^{n} \bigr) + (1-t) \Biggl(I- \kappa_{t}\sum_{i = 1}^{\infty}\omega _{i}^{(1)}W_{i} \Biggr) \Biggl(\sum _{i=1}^{\infty}\omega _{i}^{(2)}J_{r_{n,i}}^{A_{i}}(I-r_{n,i}C_{i})Q_{D}u_{t}^{n} \Biggr) \\ \qquad{} - \sum_{i = 1}^{\infty} \omega _{i}^{(2)}J^{A_{i}}_{r_{n,i}}(I-r_{n,i}C_{i}) \biggl(\frac{u_{n}+v_{n}}{2} \biggr)\Biggr\Vert ^{p} \\ \qquad{} + p \Biggl\langle \sum_{i = 1}^{\infty} \omega _{i}^{(2)}J^{A_{i}}_{r_{n,i}}(I-r_{n,i}C_{i}) \biggl(\frac{u_{n}+v_{n}}{2} \biggr) - \frac {u_{n}+v_{n}}{2}, J_{p} \biggl(u_{t}^{n} - \frac{u_{n}+v_{n}}{2} \biggr) \Biggr\rangle \\ \quad \leq \biggl\Vert u_{t}^{n}-\frac{u_{n}+v_{n}}{2} \biggr\Vert ^{p} + pt \Biggl\langle f \bigl(u_{t}^{n} \bigr) - \sum_{i = 1}^{\infty} \omega _{i}^{(2)}J^{A_{i}}_{r_{n,i}}(I-r_{n,i}C_{i})Q_{D}u_{t}^{n} \\ \qquad{} -\frac{\kappa_{t}}{t}(1-t)\sum_{i = 1}^{\infty} \omega _{i}^{(1)}W_{i} \Biggl(\sum _{i = 1}^{\infty} \omega _{i}^{(2)}J^{A_{i}}_{r_{n,i}}(I-r_{n,i}C_{i})Q_{D}u_{t}^{n} \Biggr),\\ \qquad J_{p} \Biggl(u_{t}^{n} - \sum _{i = 1}^{\infty} \omega_{i}^{(2)}J^{A_{i}}_{r_{n,i}}(I-r_{n,i}C_{i}) \biggl(\frac {u_{n}+v_{n}}{2} \biggr) \Biggr) \Biggr\rangle \\ \qquad{} +p \Biggl\langle \sum_{i = 1}^{\infty} \omega _{i}^{(2)}J^{A_{i}}_{r_{n,i}}(I-r_{n,i}C_{i}) \biggl(\frac{u_{n}+v_{n}}{2} \biggr) - \frac {u_{n}+v_{n}}{2}, J_{p} \biggl(u_{t}^{n} - \frac{u_{n}+v_{n}}{2} \biggr) \Biggr\rangle , \end{gathered} $$

which implies that

$$\begin{gathered} t \Biggl\langle \sum_{i = 1}^{\infty} \omega _{i}^{(2)}J^{A_{i}}_{r_{n,i}}(I-r_{n,i}C_{i})Q_{D}u_{t}^{n} - f \bigl(u_{t}^{n} \bigr) \\ \qquad{}+\frac{\kappa_{t}}{t}(1-t)\sum_{i = 1}^{\infty} \omega _{i}^{(1)}W_{i} \Biggl(\sum _{i = 1}^{\infty} \omega _{i}^{(2)}J^{A_{i}}_{r_{n,i}}(I-r_{n,i}C_{i})Q_{D}u_{t}^{n} \Biggr), \\ \qquad J_{p} \Biggl(u_{t}^{n} - \sum _{i = 1}^{\infty} \omega _{i}^{(2)}J^{A_{i}}_{r_{n,i}}(I-r_{n,i}C_{i}) \biggl(\frac{u_{n}+v_{n}}{2} \biggr) \Biggr) \Biggr\rangle \\ \quad\leq \Biggl\Vert \sum_{i = 1}^{\infty} \omega _{i}^{(2)}J^{A_{i}}_{r_{n,i}}(I-r_{n,i}C_{i}) \biggl(\frac{u_{n}+v_{n}}{2} \biggr) - \frac {u_{n}+v_{n}}{2} \Biggr\Vert \biggl\Vert u_{t}^{n}-\frac{u_{n}+v_{n}}{2} \biggr\Vert ^{p-1}. \end{gathered} $$

So, $\lim_{t \rightarrow0}\operatorname{limsup}_{n\rightarrow+\infty}\langle\sum_{i = 1}^{\infty} \omega_{i}^{(2)}J^{A_{i}}_{r_{n,i}}(I-r_{n,i}C_{i})Q_{D}u_{t}^{n} - f(u_{t}^{n})+\frac{\kappa_{t}}{t}(1-t)\times [4]\sum_{i = 1}^{\infty} \omega _{i}^{(1)}W_{i}(\sum_{i = 1}^{\infty} \omega _{i}^{(2)}J^{A_{i}}_{r_{n,i}}(I-r_{n,i}C_{i})Q_{D}u_{t}^{n}), J_{p}(u_{t}^{n} - \sum_{i = 1}^{\infty} \omega_{i}^{(2)}J^{A_{i}}_{r_{n,i}}(I-r_{n,i}C_{i})(\frac {u_{n}+v_{n}}{2})) \rangle \leq0$.

Since $u_{t}^{n} \rightarrow q_{0}$, then $\sum_{i = 1}^{\infty} \omega _{i}^{(2)}J^{A_{i}}_{r_{n,i}}(I-r_{n,i}C_{i})Q_{D}u_{t}^{n} \rightarrow\sum_{i = 1}^{\infty} \omega_{i}^{(2)}J^{A_{i}}_{r_{n,i}}(I-r_{n,i}C_{i})Q_{D}q_{0} = q_{0}$, as $t \rightarrow0$.

Noticing that

$$\begin{gathered} \Biggl\langle q_{0}- f(q_{0}), J_{p} \Biggl(q_{0} - \sum_{i = 1}^{\infty} \omega _{i}^{(2)}J^{A_{i}}_{r_{n,i}}(I-r_{n,i}C_{i}) \biggl(\frac{u_{n}+v_{n}}{2} \biggr) \Biggr) \Biggr\rangle \\ \quad= \Biggl\langle q_{0}-f(q_{0}), J_{p} \Biggl(q_{0} - \sum_{i = 1}^{\infty} \omega _{i}^{(2)}J^{A_{i}}_{r_{n,i}}(I-r_{n,i}C_{i}) \biggl(\frac{u_{n}+v_{n}}{2} \biggr) \Biggr) \\ \qquad - J_{p} \Biggl(u_{t}^{n} - \sum _{i = 1}^{\infty} \omega _{i}^{(2)}J^{A_{i}}_{r_{n,i}}(I-r_{n,i}C_{i}) \biggl(\frac{u_{n}+v_{n}}{2} \biggr) \Biggr) \Biggr\rangle \\ \qquad{} + \Biggl\langle q_{0} - f(q_{0}), J_{p} \Biggl(u_{t}^{n} -\sum_{i = 1}^{\infty} \omega_{i}^{(2)}J^{A_{i}}_{r_{n,i}}(I-r_{n,i}C_{i}) \biggl(\frac {u_{n}+v_{n}}{2} \biggr) \Biggr) \Biggr\rangle \\ \quad = \Biggl\langle q_{0}-f(q_{0}), J_{p} \Biggl(q_{0} - \sum_{i = 1}^{\infty} \omega _{i}^{(2)}J^{A_{i}}_{r_{n,i}}(I-r_{n,i}C_{i}) \biggl(\frac{u_{n}+v_{n}}{2} \biggr) \Biggr) \\ \qquad - J_{p} \Biggl(u_{t}^{n} - \sum _{i = 1}^{\infty} \omega _{i}^{(2)}J^{A_{i}}_{r_{n,i}}(I-r_{n,i}C_{i}) \biggl(\frac{u_{n}+v_{n}}{2} \biggr) \Biggr) \Biggr\rangle \\ \qquad{} + \Biggl\langle q_{0}-f(q_{0})- \sum _{i = 1}^{\infty} \omega _{i}^{(2)}J^{A_{i}}_{r_{n,i}}(I-r_{n,i}C_{i})Q_{D}u_{t}^{n}+f \bigl(u_{t}^{n} \bigr) \\ \qquad{} -\frac{\kappa_{t}}{t}(1-t)\sum_{i = 1}^{\infty} \omega _{i}^{(1)}W_{i} \Biggl(\sum _{i=1}^{\infty}\omega _{i}^{(2)}J_{r_{n,i}}^{A_{i}}(I-r_{n,i}C_{i})Q_{D}u_{t}^{n} \Biggr), \\ \qquad J_{p} \Biggl(u_{t}^{n} - \sum _{i = 1}^{\infty} \omega _{i}^{(2)}J^{A_{i}}_{r_{n,i}}(I-r_{n,i}C_{i}) \biggl(\frac{u_{n}+v_{n}}{2} \biggr) \Biggr) \Biggr\rangle \\ \qquad{} + \Biggl\langle \sum_{i = 1}^{\infty} \omega _{i}^{(2)}J^{A_{i}}_{r_{n,i}}(I-r_{n,i}C_{i})Q_{D}u_{t}^{n}-f \bigl(u_{t}^{n} \bigr) \\ \qquad{} +\frac{\kappa_{t}}{t}(1-t)\sum_{i=1}^{\infty} \omega _{i}^{(1)}W_{i} \Biggl(\sum _{i=1}^{\infty}\omega _{i}^{(2)}J_{r_{n,i}}^{A_{i}}(I-r_{n,i}C_{i})Q_{D}u_{t}^{n} \Biggr), \\ \qquad J \Biggl(u_{t}^{n} - \sum _{i = 1}^{\infty} \omega _{i}^{(2)}J^{A_{i}}_{r_{n,i}}(I-r_{n,i}C_{i}) \biggl(\frac{u_{n}+v_{n}}{2} \biggr) \Biggr) \Biggr\rangle , \end{gathered} $$

we have $\operatorname{limsup}_{n\rightarrow+\infty}\langle q_{0}- f(q_{0}), J_{p}(q_{0} - \sum_{i = 1}^{\infty} \omega _{i}^{(2)}J^{A_{i}}_{r_{n,i}}(I-r_{n,i}C_{i})(\frac{u_{n}+v_{n}}{2}))\rangle\leq0$.

From assumptions (iv) and (v) and Step 2, we know that $x_{n+1}-\sum_{i = 1}^{\infty} \omega_{i}^{(2)} J_{r_{n,i}}^{A_{i}}(I-r_{n,i}C_{i})(\frac {u_{n}+v_{n}}{2})\rightarrow0$ and then $\operatorname{limsup}_{n\rightarrow+\infty}\langle q_{0}- f(q_{0}), J_{p}(q_{0} - x_{n+1})\rangle\leq0$. Thus $\operatorname{limsup}_{n \rightarrow\infty}\varepsilon _{n}^{(2)}\leq0$.

Employing (2.12) again, we have

$$\Vert x_{n} - q_{0} \Vert ^{p} \leq \frac{ \Vert x_{n} - q_{0} \Vert ^{p} - \Vert x_{n+1} - q_{0} \Vert ^{p} }{\varepsilon _{n}^{(1)}}+\varepsilon_{n}^{(2)}. $$

Assumption (iv) implies that $\operatorname{liminf}_{n \rightarrow\infty}\frac{ \Vert x_{n} - q_{0} \Vert ^{p} - \Vert x_{n+1} - q_{0} \Vert ^{p} }{\varepsilon_{n}^{(1)}} = 0$. Then

$$\lim_{n \rightarrow\infty} \Vert x_{n} - q_{0} \Vert ^{p} \leq \mathop{\operatorname{liminf}}_{n \rightarrow\infty}\frac{ \Vert x_{n} - q_{0} \Vert ^{p} - \Vert x_{n+1} - q_{0} \Vert ^{p} }{\varepsilon_{n}^{(1)}}+\mathop{\operatorname{limsup}}_{n \rightarrow\infty} \varepsilon_{n}^{(2)}\leq0. $$

Then the result that $x_{n} \rightarrow q_{0}$ follows.

Case 2. If $\{ \Vert x_{n} - q_{0} \Vert \}$ is not eventually decreasing, then we can find a subsequence $\{ \Vert x_{n_{k}} - q_{0} \Vert \}$ so that $\Vert x_{n_{k}} - q_{0} \Vert \leq \Vert x_{n_{k+1}} - q_{0} \Vert $ for all $k \geq1$. From Lemma 1.7, we can define a subsequence $\{ \Vert x_{\tau (n)} - q_{0} \Vert \}$ so that $\max\{ \Vert x_{\tau(n)} - q_{0} \Vert , \Vert x_{n} - q_{0} \Vert \}\leq \Vert x_{\tau(n)+1} - q_{0} \Vert $ for all $n > n_{1}$. This enables us to deduce that (similar to Case 1)

$$0 \leq\varepsilon_{\tau(n)}^{(3)}\leq\varepsilon_{\tau (n)}^{(1)} \bigl(\varepsilon_{\tau(n)}^{(2)}- \Vert x_{\tau(n)} - q_{0} \Vert ^{p} \bigr)+ \bigl( \Vert x_{\tau(n)} - q_{0} \Vert ^{p}- \Vert x_{{\tau(n)}+1} - q_{0} \Vert ^{p} \bigr)\rightarrow0, $$

and then copying Case 1, we have $\lim_{n \rightarrow\infty} \Vert x_{\tau(n)} - q_{0} \Vert = 0$. Thus $0 \leq \Vert x_{n} - q_{0} \Vert \leq \Vert x_{\tau(n)+1} - q_{0} \Vert \rightarrow0$, as $n \rightarrow\infty$.

This completes the proof. □

### Remark 2.3

Theorem 2.2 is reasonable if we suppose $X = D = (-\infty, +\infty)$, take $f(x) = \frac{x}{4}$, $A_{i} x = C_{i} x = \frac{x}{2^{i}}$, $W_{i}x = \frac{x}{2^{i+1}}$, $\theta_{i} = 2^{i}$, $\omega_{i}^{(1)} = \omega _{i}^{(2)} = \frac{1}{2^{i}}$, $\alpha_{n} = 1-\frac{1}{n^{2}}$, $\beta_{n} = \frac{1}{n^{3}}$, $\vartheta_{n} = \delta_{n} = \frac{1}{n}$, $\xi_{n} = \zeta _{n} = a_{n} = b_{n} = \frac{1}{n^{2}}$, $\gamma_{i} = \frac{1}{2^{i+2}}$, $\mu_{i} = \frac{2^{i+1}-\frac{3}{2}+\frac{1}{2^{i+1}}}{2^{i+1}-1}$, $r_{n,i} = \frac{1}{2^{n+i}}$ for $n \in N$ and $i \in N$.

### Remark 2.4

Our differences from the main references are:

(i) the normalized duality mapping $J: E \rightarrow E^{*}$ is no longer required to be weakly sequentially continuous at zero as that in [9];
(ii) the parameter $\{r_{n,i}\}$ in the resolvent $J_{r_{n,i}}^{A_{i}}$ does not need satisfying the condition ‘$\sum_{n=1}^{\infty} \vert r_{n+1,i} - r_{n,i} \vert < +\infty$ and $r_{n,i}\geq\varepsilon > 0$ for $i \in N$ and some $\varepsilon> 0$’ as that in [3] or [9];
(iii) Lemma 1.7 plays an important role in the proof of strong convergence of the iterative sequence, which leads to different restrictions on the parameters and different proof techniques compared to the already existing similar works.

## Applications

### Integro-differential systems

In Section 3.1, we shall investigate the following nonlinear integro-differential systems involving the generalized $p_{i}$-Laplacian, which have been studied in [3]:

3.1 $$ \textstyle\begin{cases} \frac{\partial u^{(i)}(x,t)}{\partial t} -\operatorname{div}[(C(x,t)+ \vert \nabla u^{(i)} \vert ^{2})^{\frac {p_{i}-2}{2}}\nabla u^{(i)}] + \varepsilon \vert u^{(i)} \vert ^{r_{i}-2}u^{(i)}\\ \quad {}+ g(x,u^{(i)}, \nabla u^{(i)})+ a \frac{\partial}{\partial t} \int _{\Omega}u^{(i)} \,dx = f(x,t),\quad(x,t) \in\Omega\times(0,T),\\ -\langle\vartheta,(C(x,t)+ \vert \nabla u^{(i)} \vert ^{2})^{\frac{p_{i}-2}{2}}\nabla u^{(i)} \rangle\in\beta_{x}(u^{(i)}), \quad(x,t) \in\Gamma\times(0,T),\\ u^{(i)}(x,0) = u^{(i)}(x, T), \quad x \in\Omega, i \in N, \end{cases} $$

where Ω is a bounded conical domain of a Euclidean space $R^{N}$ ($N\geq1$), Γ is the boundary of Ω with $\Gamma\in C^{1}$ and *ϑ* denotes the exterior normal derivative to Γ. $\langle\cdot,\cdot\rangle$ and $\vert \cdot \vert $ denote the Euclidean inner-product and the Euclidean norm in $R^{N}$, respectively. *T* is a positive constant. $\nabla u^{(i)} = (\frac{\partial u^{(i)}}{\partial x_{1}}, \frac{\partial u^{(i)}}{\partial x_{2}}, \ldots, \frac{\partial u^{(i)}}{\partial x_{N}})$ and $x = (x_{1}, x_{2}, \ldots, x_{N}) \in\Omega$. $\beta_{x}$ is the subdifferential of $\varphi_{x}$, where $\varphi_{x}= \varphi(x,\cdot):R\rightarrow R$ for $x\in\Gamma$. *a* and *ε* are non-expansive constants, $0 \leq C(x,t) \in \bigcap_{i = 1}^{\infty}V_{i}: = \bigcap_{i = 1}^{\infty} L^{p_{i}}(0, T; W^{1,p_{i}}(\Omega))$, $f(x,t)\in\bigcap_{i = 1}^{\infty} W_{i}: = \bigcap_{i = 1}^{\infty} L^{\max\{p_{i},p_{i}'\}}(0,T; L^{\max\{p_{i},p_{i}'\}}(\Omega))$ and $g:\Omega\times R^{N+1} \rightarrow R$ are given functions.

Just like [3], we need the following assumptions to discuss (3.1).

#### Assumption 1

$\{p_{i}\}_{i=1}^{\infty}$ is a real number sequence with $\frac{2N}{N+1} < p_{i} < +\infty$, $\{\theta_{i}\}_{i=1}^{\infty}$ is any real number sequence in $(0,1]$ and $\{r_{i}\}_{i=1}^{\infty}$ is a real number sequence satisfying $\frac{2N}{N+1} < r_{i} \leq \min\{p_{i},p_{i}'\} < +\infty$. $\frac{1}{p_{i}}+\frac{1}{p'_{i}} = 1$ and $\frac{1}{r_{i}}+\frac{1}{r'_{i}} = 1$ for $i \in N$.

#### Assumption 2

Green’s formula is available.

#### Assumption 3

For each $x\in\Gamma$, $\varphi_{x}= \varphi(x,\cdot):R\rightarrow R$ is a proper, convex and lower-semicontinuous function and $\varphi_{x}(0)=0$.

#### Assumption 4

$0 \in\beta_{x}(0)$ and for each $t \in R$, the function $x \in\Gamma\rightarrow(I+\lambda\beta_{x})^{-1}(t)\in R$ is measurable for $\lambda > 0$.

#### Assumption 5

Suppose that $g:\Omega\times R^{N+1} \rightarrow R$ satisfies the following conditions:

- Carathéodory’s conditions;
- Growth condition.
  $$\bigl\vert g(x,r_{1},\ldots,r_{N+1}) \bigr\vert ^{\max\{p_{i},p_{i}'\}}\leq \bigl\vert h_{i}(x,t) \bigr\vert ^{p_{i}}+ b_{i} \vert r_{1} \vert ^{p_{i}}, $$
  where $(r_{1}, r_{2}, \ldots, r_{N+1})\in R^{N+1} $, $h_{i}(x,t)\in W_{i}$ and $b_{i}$ is a positive constant for $i \in N$;
- Monotone condition. *g* is monotone in the following sense:
  $$\bigl(g(x,r_{1},\ldots,r_{N+1})-g(x,t_{1}, \ldots,t_{N+1}) \bigr)\geq(r_{1} - t_{1}) $$
  for all $x \in\Omega$ and $(r_{1},\ldots,r_{N+1}),(t_{1},\ldots,t_{N+1})\in R^{N+1}$.

#### Assumption 6

For $i \in N$, let $V^{*}_{i}$ denote the dual space of $V_{i}$. The norm in $V_{i}$, $\Vert \cdot \Vert _{V_{i}}$, is defined by

$$\bigl\Vert u(x,t) \bigr\Vert _{V_{i}} = \biggl( \int_{0}^{T} \bigl\Vert u(x,t) \bigr\Vert _{W^{1,p_{i}}(\Omega)} ^{p_{i}}\,dt \biggr)^{\frac{1}{p_{i}}}, \quad u(x,t) \in V_{i}. $$

#### Definition 3.1

[3]

For $i \in N$, define the operator $B_{i}: V_{i} \rightarrow V^{*}_{i}$ by

$$\langle w,B_{i}u \rangle= \int_{0}^{T} \int_{\Omega}\bigl\langle \bigl(C(x,t)+ \vert \nabla u \vert ^{2} \bigr)^{\frac{p_{i}-2}{2}}\nabla u, \nabla w \bigr\rangle \,dx \,dt + \varepsilon \int_{0}^{T} \int_{\Omega} \vert u \vert ^{r_{i}-2}uw \,dx \,dt $$

for $u,w \in V_{i}$.

#### Definition 3.2

[3]

For $i \in N$, define the function $\Phi_{i}: V_{i} \rightarrow R $ by

$$\Phi_{i}(u)= \int_{0}^{T} \int_{\Gamma}\varphi_{x} \bigl(u\vert_{\Gamma}(x,t) \bigr)\,d\Gamma(x)\,dt $$

for $u(x,t) \in V_{i}$.

#### Definition 3.3

[3]

For $i \in N$, define $S_{i}: D(S_{i}) = \{u(x,t) \in V_{i}: \frac{\partial u }{\partial t} \in V^{*}_{i}, u(x,0) = [4] u(x,T)\} \rightarrow V^{*}_{i}$ by

$$S_{i}u = \frac{\partial u }{\partial t}+ a\frac{\partial}{\partial t} \int_{\Omega}u \,dx. $$

#### Lemma 3.4

[3]

*For* $i \in N$, *define a mapping* $A_{i}: W_{i} \rightarrow2^{W_{i}}$ *as follows*:

$$D(A_{i})= \bigl\{ u \in W_{i} \vert \textit{there exists an }f \in W_{i}\textit{ such that }f \in B_{i}u + \partial \Phi_{i}(u) + S_{i}u \bigr\} , $$

*where* $\partial\Phi_{i}: V_{i}\rightarrow V^{*}_{i}$ *is the subdifferential of* $\Phi _{i}$. *For* $u \in D(A_{i})$, *we set* $A_{i}u =\{f\in W_{i} \vert f \in B_{i}u + \partial \Phi_{i}(u) + S_{i}u\}$. *Then* $A_{i}: W_{i} \rightarrow2^{W_{i}}$ *is m*-*accretive*, *where* $i \in N$.

#### Lemma 3.5

[3]

*Define* $C_{i}: D(C_{i}) = L^{\max\{p_{i},p'_{i}\}}(0,T;W^{1,\max\{p_{i},p'_{i}\}}(\Omega))\subset W_{i} \rightarrow W_{i}$ *by*

$$(C_{i}u) (x,t) = g(x,u,\nabla u)-f(x,t) $$

*for* $\forall u(x,t) \in D(C_{i})$ *and* $f(x,t)$ *is the same as that in* (3.1), *where* $i \in N$. *Then* $C_{i}: D(C_{i})\subset W_{i} \rightarrow W_{i}$ *is continuous and strongly accretive*. *If we further assume that* $g(x,r_{1},\ldots,r_{N+1}) \equiv r_{1}$, *then* $C_{i}$ *is* $\theta_{i}$-*inversely strongly accretive*, *where* $i \in N$.

#### Lemma 3.6

[3]

*For* $f(x,t)\in\bigcap_{i = 1}^{\infty}W_{i}$, *integro*-*differential systems* (3.1) *have a unique solution* $u^{(i)}(x,t) \in W_{i}$ *for* $i \in N$.

#### Lemma 3.7

[3]

*If* $\varepsilon\equiv0$, $g(x,r_{1},\ldots,r_{N+1}) \equiv r_{1}$ *and* $f(x,t) \equiv k $, *where* *k* *is a constant*, *then* $u(x,t)\equiv k$ *is the unique solution of integro*-*differential systems* (3.1). *Moreover*, $\{u(x,t)\in\bigcap_{i = 1}^{\infty }W_{i}\vert u(x,t)\equiv k \textit{ satisfying }\text{(3.1)}\} = \bigcap_{i = 1}^{\infty}N(A_{i}+C_{i})$.

#### Remark 3.8

[3]

Set $p:= \inf_{i \in N}(\min\{ p_{i},p_{i}'\})$ and $q:= \operatorname{sup}_{i \in N}(\max\{p_{i},p_{i}'\})$.

Let $X:= L^{\min\{p,p'\}}(0,T; L^{\min\{p,p'\}}(\Omega))$, where $\frac{1}{p}+\frac{1}{p'} = 1$.

Let $D:= L^{\max\{q,q'\}}(0,T; W^{1,\max\{q,q'\}}(\Omega))$, where $\frac{1}{q}+\frac{1}{q'} = 1$.

Then $X = L^{p}(0,T;L^{p}(\Omega))$, $D = L^{q}(0,T;W^{1,q}(\Omega))$ and $D \subset W_{i} \subset X$, $\forall i \in N$.

#### Theorem 3.9

*Let* *D* *and* *X* *be the same as those in Remark* 3.8. *Suppose* $A_{i}$ *and* $C_{i}$ *are the same as those in Lemmas* 3.4 *and* 3.5, *respectively*. *Let* $f: X\rightarrow X$ *be a fixed contractive mapping with coefficient* $k \in(0,1)$ *and* $W_{i}: X \rightarrow X$ *be* $\mu_{i}$-*strictly pseudo*-*contractive mappings and* $\gamma_{i}$-*strongly accretive mappings with* $\mu_{i}+\gamma_{i} > 1$ *for* $i\in N$. *Suppose that* $\{\omega_{i}^{(1)}\}$, $\{\omega_{i}^{(2)}\}$, $\{\alpha_{n}\}$, $\{ \beta_{n}\}$, $\{\vartheta_{n}\}$, $\{\nu_{n}\}$, $\{\xi_{n}\}$, $\{\delta_{n}\} $, $\{\zeta_{n}\}$, $\{r_{n,i}\}$, $\{a_{n}\}\subset X$ *and* $\{b_{n}\} \subset D$ *satisfy the same conditions as those in Theorem* 2.2, *where* $n \in N$ *and* $i \in N$. *Let* $\{x_{n}\}$ *be generated by the following iterative algorithm*:

3.2 $$\begin{aligned} \textstyle\begin{cases} x_{1} \in D,\\ u_{n} = Q_{D}(\alpha_{n} x_{n}+\beta_{n} a_{n}),\\ v_{n} = \vartheta_{n} u_{n}+\nu_{n}\sum_{i = 1}^{\infty}\omega _{i}^{(2)}J^{A_{i}}_{r_{n,i}}(I-r_{n,i}C_{i})(\frac{u_{n}+v_{n}}{2})+\xi_{n} b_{n},\\ x_{n+1}= \delta_{n} f(x_{n})+(1-\delta_{n})(I-\zeta_{n}\sum_{i = 1}^{\infty }\omega_{i}^{(1)}W_{i})\sum_{i = 1}^{\infty}\omega _{i}^{(2)}J^{A_{i}}_{r_{n,i}}(I-r_{n,i}C_{i})(\frac{u_{n}+v_{n}}{2}),\quad n \in N.\hspace{-20pt} \end{cases}\displaystyle \end{aligned}$$

*If*, *in integro*-*differential systems* (3.1), $\varepsilon\equiv0$, $g(x,r_{1},\ldots,r_{N+1})\equiv r_{1}$ *and* $f(x,t)\equiv k$, *then under the following assumptions in Theorem* 2.2, *the iterative sequence* $x_{n} \rightarrow q_{0}\in\bigcap_{i = 1}^{\infty }N(A_{i}+C_{i})$, *which is the unique solution of integro*-*differential systems* (3.1) *and which satisfies the following variational inequality*: *for* $\forall y \in \bigcap_{i=1}^{\infty}N(A_{i}+C_{i})$,

$$\bigl\langle (I - f)q_{0}(x,t), J \bigl(q_{0}(x,t) - y \bigr) \bigr\rangle \leq0. $$

### Convex minimization problems

Let *H* be a real Hilbert space. Suppose $h_{i}: H \rightarrow(-\infty, +\infty)$ are proper convex, lower-semicontinuous and nonsmooth functions [2], suppose $g_{i}: H \rightarrow(-\infty, +\infty)$ are convex and smooth functions for $i \in N$. We use $\nabla g_{i}$ to denote the gradient of $g_{i}$ and $\partial h_{i}$ the subdifferential of $h_{i}$ for $i \in N$.

The convex minimization problems are to find $x^{*} \in H$ such that

3.3 $$ h_{i} \bigl(x^{*} \bigr)+g_{i} \bigl(x^{*} \bigr) \leq h_{i}(x)+g_{i}(x),\quad i \in N, $$

for $\forall x \in H$.

By Fermats’ rule, (3.3) is equivalent to finding $x^{*} \in H$ such that

3.4 $$ 0 \in\partial h_{i} \bigl(x^{*} \bigr)+\nabla g_{i} \bigl(x^{*} \bigr),\quad i \in N. $$

#### Theorem 3.10

*Let* *H* *be a real Hilbert space and* *D* *be the nonempty closed convex sunny non*-*expansive retract of* *H*. *Let* $Q_{D}$ *be the sunny non*-*expansive retraction of* *H* *onto* *D*. *Let* $f: H \rightarrow H$ *be a contraction with coefficient* $k \in(0,1)$. *Let* $h_{i}: H \rightarrow(-\infty, +\infty)$ *be proper convex*, *lower*-*semicontinuous and nonsmooth functions and* $g_{i}: H \rightarrow (-\infty, +\infty)$ *be convex and smooth functions for* $i \in N$. *Let* $W_{i}: H \rightarrow H$ *be* $\mu_{i}$-*strictly pseudo*-*contractive mappings and* $\gamma_{i}$-*strongly accretive mappings with* $\mu_{i}+\gamma_{i} > 1$ *for* $i \in N$. *Suppose* $\{\omega_{i}^{(1)}\}$, $\{\omega_{i}^{(2)}\}$, $\{\alpha_{n}\}$, $\{ \beta_{n}\}$, $\{\vartheta_{n}\}$, $\{\nu_{n}\}$, $\{\xi_{n}\}$, $\{\delta_{n}\} $, $\{\zeta_{n}\}$, $\{r_{n,i}\}\subset(0,+\infty)$, $\{a_{n}\}\subset H$ *and* $\{b_{n}\}\subset D$ *satisfy the same conditions as those in Theorem* 2.2, *where* $n \in N$ *and* $i \in N$. *Let* $\{x_{n}\}$ *be generated by the following iterative algorithm*:

3.5 $$\begin{aligned} \textstyle\begin{cases} x_{1} \in D,\\ u_{n} = Q_{D}(\alpha_{n} x_{n}+\beta_{n} a_{n}),\\ v_{n} = \vartheta_{n} u_{n}+\nu_{n}\sum_{i = 1}^{\infty}\omega _{i}^{(2)}J^{\partial h_{i}}_{r_{n,i}}(I-r_{n,i}\nabla g_{i})(\frac {u_{n}+v_{n}}{2})+\xi_{n} b_{n},\\ x_{n+1}= \delta_{n} f(x_{n})+(1-\delta_{n})(I-\zeta_{n}\sum_{i = 1}^{\infty }\omega_{i}^{(1)}W_{i})\sum_{i = 1}^{\infty}\omega_{i}^{(2)}J^{\partial h_{i}}_{r_{n,i}}(I-r_{n,i}\nabla g_{i})(\frac{u_{n}+v_{n}}{2}),\quad n \in N.\hspace{-20pt} \end{cases}\displaystyle \end{aligned}$$

*If*, *further*, *suppose* $\nabla g_{i}$ *is* $\frac{1}{\theta_{i}}$-*Lipschitz continuous and* $h_{i}+g_{i}$ *attains a minimizer*, *then* $\{x_{n}\}$ *converges strongly to the minimizer of* $h_{i}+g_{i}$ *for* $i\in N$.

#### Proof

It follows from [2] that $\partial h_{i}$ is m-accretive. From [19], since $\nabla g_{i}$ is $\frac{1}{\theta_{i}}$-Lipschitz continuous, then $\nabla g_{i}$ is $\theta_{i}$-inversely strongly accretive. Thus Theorem 2.2 ensures the result.

This completes the proof. □
